# Importance of Polymorphisms in the Gene of Paraoxonase-1 (SNP rs662) and Apolipoprotein A-I (SNP rs670 and rs5069) in Non-Smoking and Smoking Healthy Subjects and Patients with Acute Pancreatitis

**DOI:** 10.3390/genes13111968

**Published:** 2022-10-28

**Authors:** Milena Ściskalska, Halina Milnerowicz

**Affiliations:** 1 Division of Biomedical and Environmental Analyses, Department of Pharmaceutical Biochemistry, Faculty of Pharmacy, Wroclaw Medical University, 211A Borowska St., 50-556 Wroclaw, Poland; 2 Department of Biomedical and Environmental Analyses, Faculty of Pharmacy, Wroclaw Medical University, Borowska 211 St., 50-556 Wroclaw, Poland

**Keywords:** apolipoprotien A-I, high density lipoprotein, HDL, acute pancreatitis, paraoxonase-1, PON1, rs662, rs670, rs5069, smoking

## Abstract

Oxidative stress has been implicated in the initiation of acute pancreatitis (AP). HDL is considered to be a preventing factor against cell membrane oxidation, thanks to the presence on its surface of apolipoprotein A-I (apoA-I) and paraoxonase-1 (PON1), which activity can be modified by genetic and environmental factors. The impact of SNP rs662 in the *PON1* gene and SNP rs670 and rs5069 in the *APOAI* gene on PON1 activities and its concentration in the population of AP patients and healthy volunteers was investigated. In the group of patients with AP, a decreased HDL concentration and PON1 activities were observed. A decrease in the aryloesterase and lactonase activities of PON1 in AP patients with the TT genotype for SNP rs662 (especially in smokers) was found. In the group of patients with the AA genotype (rs670), the highest concentrations of HDL and apoA-I were observed, which were gradually decreasing in the course of AP. Changes in the concentration of apoA-I were associated with the changes in the concentration and activities of PON1 in the AP patients with the AA genotype for SNP rs670. A decreasing apoA-I concentration contributing to lowering PON1 concentration and its activities during the hospitalization of AP patients with the CC genotype for SNP rs5069 were shown. Therefore, more susceptibility of persons with the CC genotype for SNP rs5069 to pro/antioxidative imbalance was shown. In this process, an important role was played by the HDL level and its interaction with PON1 and apoA-I.

## 1. Introduction

Acute pancreatitis (AP) is a severe illness representing an important challenge for physicians; it is responsible for ~3% mortality for AP cases with interstitial (oedematous) type and 30–40% among hospitalized patients with pancreatitis and organ failure or pancreatic necrosis [1,2]. According to the data reported in 2022, it has an incidence of 4.6–100/100,000 persons in Europe and is increasing over time [1,2]. The main aetiologies of acute pancreatitis are gallstones, pancreatic ischemia, or excessive consumption of alcohol [2]. Other notable causes are metabolic (hypercalcemia, hypertriglyceridemia), drug-induced, post–endoscopic retrograde cholangiopancreatography, autoimmune, infectious, trauma, congenital, genetic, and idiopathic [2]. The activation of inflammatory cytokines and pancreatic proteases is considered a major pathophysiologic process in AP [1]. The AP occurrence is a result of an intense inflammatory response as a consequence of the imbalance between anti-inflammatory mechanisms and pro-inflammatory mediators [1]. It was reported that free radicals play a significant role in the initiation of inflammation of the pancreas with poor antioxidant status [1]. Increased levels of free radicals can cause cell damage directly by the destruction of the cell membrane, via the toxicity of lipids peroxidation products, or altered signalling pathways, including redox regulation of genes [1].

The course of AP contributes to lipid profile modification, which is reflected in decreased high-density lipoprotein (HDL) concentration, considered an independent predictor for organ failure, pancreatic necrosis, and mortality in AP [3]. HDL is considered a preventing factor against cell membrane oxidation induced by pro/antioxidative imbalance [4,5]. This property of HDL is attributed to the presence on its surface of a specific enzyme—paraoxonase-1 (PON1, EC 3.1.8.1) [4,6]. Human PON1 (43–45 kDa) is a membrane-bound glycoprotein that included 354 amino acids [5,7]. PON1 is a member of the paraoxonase multi-gene family which comprises three members, PON1, PON2, and PON3 [5,6]. PON1 is expressed in a variety of tissues but is mainly synthesized by the liver [4,7]. This multifunctional glycoprotein has significant anti-oxidative and anti-inflammatory properties through its enzymatic lactonase, paraoxonase, and esterase activities [5,7,8,9]. This enzyme is an effective xenobiotic metabolizer [10]. It was reported that high variability in PON1 activity has been attributed to genetic and environmental factors [4,5,6]. It was shown that the rate of hydrolysis of some substrates by the isoforms of the PON1 enzyme is determined by single nucleotide polymorphisms (SNPs), among others SNP rs662 present in the coding region [5,7]. This SNP is based on amino acid substitution from glutamine (Q) to arginine (R) at codon 192 (Q192R) resulting in the exchange of codon CAA to CGA in exon six of the *PON1* gene [4,5,7].

PON1 is associated with apolipoprotein A-I (apoA-I) in the HDL particles [10]. ApoA-I, the major protein constituent of HDL, is widely known for regulating cholesterol trafficking and the maturation of HDL particles [11,12]. It may also modulate inflammatory and immune responses [12]. ApoA-I is a key protein of HDL, essential for binding to the PON1 molecule [13]. The *APOA1* gene located on chromosome 11q23-q24, is highly polymorphic [11,14]. In this gene, several SNPs have been identified to influence serum lipoprotein concentrations [11]. Among these, a common G/A substitution located at –75 bp upstream from the *APOA1* gene transcription start site (rs670) and a C/T substitution at position +83 bp (rs5069) from the first intron were shown to differentially affect the apoA-I expression and lipids concentration [11,14,15].

Our previous study has shown that the course of AP is associated with a decreased HDL concentration accompanied by decreased PON1 activities [16]. It was also shown that environmental factors such as exposure to tobacco smoke xenobiotics have an effect on PON1 activities [16]. The aim of this study was to assess whether the observed reduction in PON1 activities was only due to HDL lowering or whether other factors, such as the apoA-I concentration and genetic variables, can also influence PON1 activities in the course of AP. The study was focused on investigating the variability in the activities of PON1 (phosphotriesterase, arylesterase, and lactonase activity) and its concentration in the population of patients with acute pancreatitis (AP) and healthy volunteers exposed to tobacco smoke xenobiotics in terms of genotypes for SNP rs662 in the *PON1* gene. The concentrations of apoA-I and HDL as an important particle affecting PON1 activity were measured. The parameters describing the disorders in the lipid profile (the value of ratios: TC/HDL, LDL/HDL) were determined. The concentration of malonyldialdehyde (MDA) and the value of oxLDL/LDL ratio as a markers of oxidative stress induced by inflammation were also assessed. The other aim of this study was to check an influence of genotypes for SNP rs670 and rs5069 in *APOAI* gene on above mentioned parameters. Additionally, the activities and concentration of PON1, the concentration of apoA-I, HDL, the value of TC/HDL, LDL/HDL, oxLDL/LDL ratios and MDA concentration were analysed during the hospitalization of AP patients. The relation of SNP rs662 in the *PON1* gene, rs670 and rs5069 in *APOAI* gene with the risk of AP occurrence were also assessed.

## 2. Materials and Methods

### 2.1. Study Population

A total of 95 individuals were included in the study. Patients and controls were a homogeneous group who resided in Poland. All individuals received full and thorough information about the study. All participants provided informed written consent before they were included in the study. The study was carried out in accordance with the Declaration of Helsinki and the design of the study was approved by the Commission of Bioethics at Wroclaw Medical University (KB-529/2018 and KB-215/2020).

The control group included 51 healthy volunteers. The qualification of healthy subjects to the study was performed by primary care physicians based on the questionnaire and physical examination. The 44 patients with AP were enrolled from the Second Clinic of General and Oncological Surgery of Wroclaw Medical University (Wroclaw, Poland). Diagnosis of AP was confirmed according to finding at least two of the following criteria: acute onset of a persistent, severe, epigastric pain characteristic for AP; three-fold elevation of serum lipase or amylase, or elevation above the upper limit of reference range; characteristic findings of acute pancreatitis on imaging (transabdominal ultrasonography, contrast-enhanced computed tomography (CT) or magnetic resonance imaging). The etiology of AP in individuals enrolled in the study was as follows: biliary (27.3%), alcohol (18.1%), hyperlipidaemia (16%), alcohol and hyperlipidaemia (18.1%), and other causes (20.5%). During treatment, an intensive intravenous fluid and low-fat diet monitored by symptoms resolution and laboratory test improvement were introduced, as described earlier [17,18]. The exclusion criteria consisted of accompanying diseases, such as cancer, diabetes, liver diseases, cardiovascular diseases, chronic inflammatory diseases other than AP, and simultaneous treatment with more than two types of drugs, regardless of their mechanism of action.

In the study population, a personal interview about anthropometric variables and lifestyle (nutritional habits, any use of medications/dietary supplements, frequency of alcohol intake, and smoking history) was conducted. The data about the number of cigarettes smoked per day, duration of smoking, smoking cessation, the occurrence of smoking-related diseases, and passive exposure to cigarette smoke were obtained and used to assess the intensity of the exposition to cigarette smoke. This information was verified by the determination of serum concentration of cotinine—a major nicotine metabolite, on the basis of which the subjects were categorized as non-smokers and smokers. The cotinine cut-off value for smokers was established at 10 ng/mL. The persons with cotinine concentrations less than 10 ng/mL were considered non-smokers. The characteristic of healthy subjects and AP patients in Table 1 was shown.

Biochemical measurements were determined in the venous blood collected by venipuncture overnight (>12 h), but no later than 24 h after the first symptoms of AP. The blood samples from AP patients were taken on their first, third, and seventh days of hospitalization. The blood samples of healthy subjects were obtained from the biobank of the Polish Center for Technology Development (Wroclaw, Poland).

The biochemical parameters were measured in serum and plasma collected from participants included to study population. Venous blood samples were collected into disposable trace-element-free tubes (Cat. no.: 368815, Becton Dickinson, Germany) with serum clotting activator for preparation of serum, left at 25 °C to complete thrombosis, and centrifuged (1200 g, 20 min). To obtain plasma, the blood was drawn into tubes containing heparin (Cat. no.: 368886, Becton Dickinson, Heidelberg, Germany) and centrifuged (2500× *g*, 15 min). Plasma samples were separated from buffy coat and erythrocyte pellet. The collected samples of serum and plasma were portioned and stored in sealed tubes (Cat. no.: 0030102.002, Eppendorf, Hamburg, Germany). Part of the obtained serum samples was used for determinations performed on fresh biological material and the remainder was frozen and stored at −25 °C until analysis along with samples of plasma.

The buffy coat layer (for DNA isolation) was obtained from whole blood collected into tubes containing disodium EDTA (Cat No.: 367864, Becton Dickinson, Germany) as described earlier [19]. Blood samples were centrifuged (2.500× *g*, 15 min) to separate plasma and erythrocyte pellets. The buffy coat was removed from the erythrocyte pellet and it was transferred to a fresh tube, resuspended, and washed in phosphate-buffered saline (PBS), then centrifuged (16,000× *g*, 3 min) to remove the PBS. The samples of the buffy coat were stored at −80 °C until analysis.

### 2.2. Methods

To determine the concentration of cotinine in serum, the Cotinine ELISA test (Cat. No.: EIA-3242, DRG International, Inc., Springfield, NJ, USA) was used.

Phosphotriesterase activity of PON1 (PON1(P)) was assayed in fresh serum using synthetic paraoxon (Cat. No.: 311-45-5; Sigma-Aldrich, Schnelldorf, Germany) as substrate. This method is based on measuring the initial rate of paraoxon hydrolysis to *p*-nitrophenol, the amount of which was monitored by the change in absorbance over time at λ = 405 nm as described earlier [16]. The assay was performed at 37 °C in a 100 mM Tris-HCl buffer (pH 8.5) containing 2 mM CaCl_2_. The enzyme activity was calculated from the molar extinction coefficient of *p*-nitrophenol (18.053 (μmol/L)^−1^ cm^−1^). One unit of PON1(P) activity was expressed as 1 μmol of paraoxon hydrolyzed per minute at a temperature of 37 °C.

Arylesterase activity of PON1 (PON1(A)) was assayed in fresh serum using phenyl acetate (Cat. No.: 122-79-2; Sigma Aldrich, Germany) as a substrate, according to the procedure described earlier [16]. The method utilises the hydrolysis of phenyl acetate and formation of phenol, which resulted in a change in absorbance at λ = 270 nm over time. This assay was performed at 37 °C in a 20 mM Tris-HCl buffer (pH 8.0) containing 1 mM CaCl_2_. The PON1(A) activity was calculated from the molar extinction coefficient of phenol (1310 (mol/L)^−1^ cm^−1^). One unit of PON1(A) activity was expressed as 1 μmol of phenyl acetate hydrolyzed per minute at a temperature of 37 °C.

Lactonase activity of PON1 (PON1(L)) was measured in fresh serum using dihydrocoumarin (Cat. No.: 119-84-6; Sigma-Aldrich, Germany) as a substrate, as described previously [16]. This method is based on the hydrolysis of substrate and formation of 3-(2-hydroxyphenyl)propionate at 37 °C in a 50 mM Tris-HCl buffer (pH 7.0) containing 1 mM CaCl_2_. The amount of the resulting reaction product was measured by a change in absorbance over time at λ = 270 nm. The PON1(L) activity was calculated from the molar extinction coefficient of 3-(2-hydroxyphenyl)propionate (1870 (mol/L)^−1^ cm^−1^). One unit of PON1(L) activity was expressed as 1 μmol of dihydrocoumarin hydrolyzed per minute at a temperature of 37 °C.

The changes in absorbance for PON1 activities (PON1(P), PON1(A), and PON1(L)) were measured at 10 s intervals for 1–3 min using Specord 40 (Analytic Jena, DE, Cat. No.: 400280).

To determine the concentration of PON1 in serum, the commercial Paraoxonase 1 Human ELISA kit (Cat. No.: RD191279200R, BioVendor, Brno-Řečkovice a Mokrá Hora, Czech Republic) was used.

The concentration of apoA-I was measured in serum using Apolipoprotein A1 (ApoA1) ELISA Kit (Cat. No.: 80702, Crystal Chem, Elk Grove Village, IL, USA).

HDL concentration was estimated in serum by the precipitation method using a commercially available test (Cat. No.: 1-029-0200, BioMaxima, Lublin, Poland). The method is based on the selective precipitation of chylomicrons, low-density lipoproteins (LDL), and very low-density lipoproteins (VLDL) by phosphotungstic acid and magnesium chloride (MgCl_2_) followed by centrifugation. HDL, as residual cholesterol remaining in clear supernatant, was subjected to enzymatic reaction using a quantitative determination of total cholesterol.

Total cholesterol (TC) in serum was measured enzymatically with the spectrophotometric method using a set of reagents (Cat. No.: 1-023-0200; BioMaxima, Poland). Cholesterol was measured according to the procedure provided by the manufacturer. The absorbance of forming colour reaction product (proportional to cholesterol concentration) was measured at λ = 500 nm at 25 °C.

To determine the concentration of triglycerides in the serum, a commercial reagent (Cat. No.: 1-053-0200; BioMaxima, Poland) was used. Triglycerides were hydrolyzed by lipase to produce glycerol, which was oxidised using oxidase. One of the reaction products was H_2_O_2_, which reacted with the 4-aminoantipyrine resulting in colour product. The absorbance was measured at λ = 500 nm at 25 °C.

The concentration of low-density lipoproteins (LDL) was calculated using the Friedewald formula.

The concentration of oxLDL in the serum was determined using Mercodia Oxidised LDL ELISA kit (Cat.: No.: 10-1143-01, Mercodia, Uppsala, Sweden).

To measure the MDA concentration in plasma, the colorimetric method using Lipid Peroxidation (MDA) Assay Kit (Cat. No. MAK085-1KT, Sigma-Aldrich, Schnelldorf, Germany) was used.

The absorbance of samples was measured using and Genesys 10S (Cat. No.: 840-208100, Thermo Scientific, Waltham, MA, USA) and MultiScan Go (Cat. No.: N10588, Thermo Scientific, USA).

#### DNA Analyses

Genomic DNA was extracted from buffy coat samples using a commercial kit (Syngen Blood/Cell DNA Mini Kit, Ref. no.: SY221012, Syngen, Biotech, Kraków, Poland). The concentration of extracted DNA was assessed by the spectrophotometric measurement samples at λ = 260 nm using µDrop Plate (Cat. no.: N12391, Thermo Scientific). The 260/280 ratio was used as the purity indicator of the DNA. The SNP rs662 in the *PON1* gene (National Center for Biotechnology Information, NCBI Reference Sequence: NG_008779.2), SNP rs670 (−75 G/A), and rs5069 (+83 C/T) in the *APOA1* gene (NCBI Reference Sequence: NG_012021.1) were identified with the polymerase chain reaction-restriction fragment length polymorphism (PCR-RFLP) method. All cases were genotyped for SNP rs662 in the *PON1* gene using the forward primer 5′ CACGAAGGCTCCATCCCAC 3′ and the reverse primer 5′ TCCTTCTGCCACCACTCGAAC 3′ (Custom DNA Oligos, Cat. No: CDO, Syngen, Biotech, Poland). PCR for genotyping of SNP rs670 and rs5069 in the *APOA1* gene was carried out using the following primers: 5′ AGGGACAGAGCTGATCCTTGAACTCTTAAG 3′ (forward) and 5′ TTAGGGGACACCTACCCGTCAGGAAGAGCA 3′(reverse). The PCR reaction mixture contained 0.6 µL of each forward and reverse primer (10 pmol/µL), 2 µL of extracted DNA, 12.8 µL of molecular grade water, and 4.0 µL of Gold Hot Start PCR Mix (Cat. no.: SY550231, Syngen) with Taq polymerase, reaction buffer, MgCl_2_, 10× modifier GC, dNTP (final volume of PCR reaction mixture was 20.0 µL). Cycling conditions were as follows: initial denaturation at 95 °C for 15 min followed by 35 cycles of 40 s at 94 °C, 35 s at 48 °C (for genotyping SNP rs662 in the *PON1* gene) or 35 s at 70 °C (for genotyping SNP rs670 and rs5069 in the *APOA1* gene), 40 s at 72 °C and a final extension time of 15 min at 72 °C. The PCR products were digested with Alw I (Cat. No.: ER1321, Thermo Fischer Scientific, Waltham, MA, USA) and MspI (Cat. No.: ER0541, Thermo Fischer Scientific, USA) for genotyping the SNP rs662 in the *PON1* gene and SNP rs670 and rs5069 in the *APOA1* gene, respectively, subsequently at 37 °C for 16 h for each enzyme, whereafter that enzymes were inactivated at 80 °C for 20 min. The agarose gel (3.0%, Cat. no.: SY521011) electrophoresis with Green DNA Gel Stain (Cat. no.: SY521032, Syngen) was performed to separate digested fragments, then visualized under UV light.

### 2.3. Statistical Analyses

The data were presented as mean ± standard deviation (X ± SD) and the 1st quartile (Q1), median, and third quartile (Q3). The Shapiro–Wilk test was used to verify the normality of the variables. To compare the two groups, the parametric Student’s *t*-test or a nonparametric Mann–Whitney U test was used. Differences between three or more groups were tested with one-way ANOVA (two-way Analysis of Variance) on ranks and Duncan’s post hoc test. Categorical variables (genotype frequency) were compared using the χ^2^ test and Fisher’s exact test. To determine the independent effect of polymorphism genotypes on the risk of disease, logistic regression tests were used. The association was measured by the odds ratio (OR) with its confidence interval (CI). Spearman’s correlation coefficient was calculated to verify correlations between the examined parameters. The *p*-value < 0.05 was considered as a threshold for statistical significance. Data analyses were carried out using Statistica 13.3 (Polish version; StatSoft, Kraków, Poland) under Wroclaw Medical University’s license.

## 3. Results

### 3.1. Intersexual Variability in the Activity of PON1, the Concentration of Lipid Profile Parameters and Selected Markers of Oxidative Stress

A lower HDL concentration was noted in the blood of healthy men compared to healthy women (Table 2). However, no intersexual difference in the HDL concentration was found in the group of AP patients. It was also shown no intersexual differences in the concentration of apoA-I and the activities of PON1 (PON(P), PON1(A), PON1(L)), and its concentration in the blood of women and men in both examined groups (healthy subjects and AP patients). Similarly, there were no differences in the value of the TC/HDL ratio, LDL/HDL, and oxLDL/LDL ratios in the group of healthy subjects and AP patients (Table 2).

The activity of PON1(P) in the blood of AP patients was noted to be lower than in healthy subjects (*p* < 0.0001 and *p* = 0.0003 for women and men, respectively), which was accompanied by lower HDL concentrations (*p* = 0.0018 and *p* = 0.0307 for women and men, respectively) (Table 2). Additionally, it was observed a lower PON(L) activity in the female AP patients compared to the appropriate group of healthy subjects (*p* = 0.0134). Our study has revealed a lower apoA-I concentration in the blood of AP patients in comparison to healthy subjects in the male individuals (*p* = 0.0005) (Table 2).

### 3.2. Results of Genotyping

**Figure 1:**
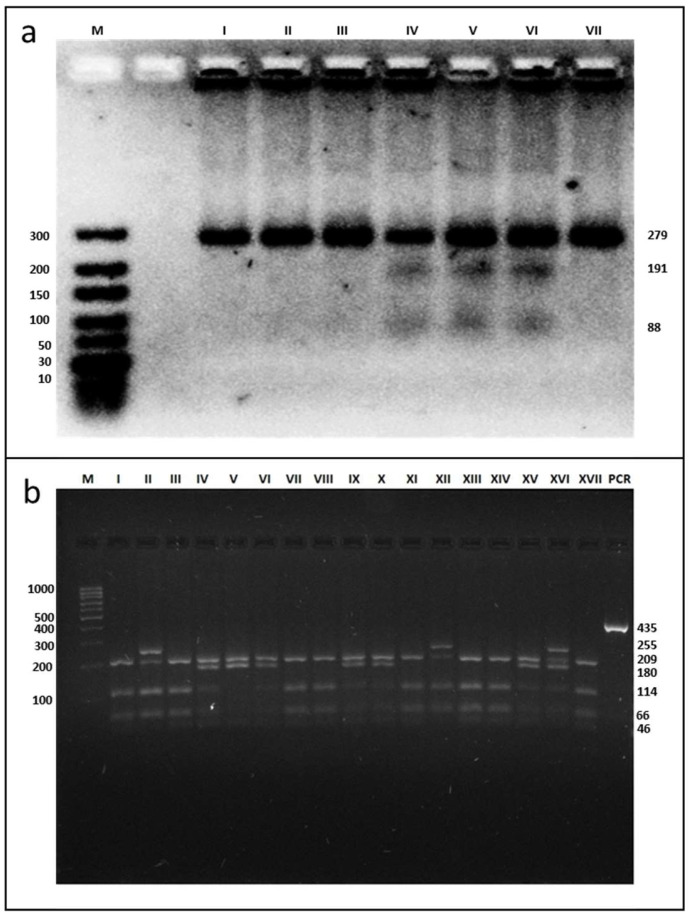
Electrophoresis pattern of *PON1* rs662 (**a**) and *APOA1* −75 bp and +83 bp (**b**) polymorphism using PCR—RFLP method. (**a**) M—marker ladder (10–300 bp); I–III, VII—TT genotype, IV–VI—TC genotype; (**b**) M—marker ladder (100–1000 bp); I, III, VII, VIII, XI, XIII, XIV, XVII—GG genotype for SNP −75 bp and the CC genotype for SNP +83 bp; II, XII—GG genotype for SNP −75 bp and the TC genotype for SNP +83 bp; IV, VI, IX, X, XV—GA genotype for SNP −75 bp and the CC genotype for SNP +83 bp; XVI—GA genotype for SNP −75 bp and the TC genotype for SNP +83 bp; V—AA genotype for SNP −75 bp and the CC genotype for SNP +83 bp; PCR—undigested PCR products.

The examined parameters were analysed for SNP rs662 in the *PON1* gene and SNP −75 bp and SNP +83 bp in the *APOA1* gene. The PCR amplification yielded a product of 279-bp for SNP rs662 in the *PON1* gene, which could be seen in all samples. Following incubation with restriction enzyme AlwI, gel electrophoresis yielded two bands: 191- and 88-bp (presence of the restriction site) for the CC homozygous. The TC genotype was characterised by a band at 279-, 191-, and 88-bp (the presence and absence of the restriction site), and the TT genotype was identified as a band at 279-bp (absence of the restriction site) (Figure 1a). The presence of the MspI restriction site at −75 bp (G allele) and at +83 bp (C allele) in the 435-bp product for SNPs analyses in the *APOA1* gene yielded four fragments: 46-, 66-, 114-, and 209-bp. The absence of the restriction site at −75 bp (A allele) resulted in three fragments of 46-, 180-, and 209-bp. The absence of the restriction site at +83 bp (T allele) created a fragment of 255-bp (instead of two fragments of 46- and 209-bp) (Figure 1b).

The frequency of genotype occurrence for the examined SNPs in the study population in Table 3 was presented. In both examined groups, healthy subjects and AP patients, homozygous TT was the most common genotype for SNP rs662 in the *PON1* gene and the frequencies of the occurrence of this genotype in the above-mentioned groups were similar. However, it was noted that the TC genotype appeared much less often in the study population. There were no cases with the CC genotype for SNP rs662 in the *PON1* gene in examined groups. It was noted that the GG genotype for SNP −76 bp and the CC genotype for SNP +83 bp in the *APOA1* gene were the most common genotypes for these SNP in the study population. The AA genotype for SNP −76 bp in the *APOA1* gene appeared the least often among the healthy subjects, same as the AG genotype for this SNP in the AP patients. There were no cases with the TT genotype for SNP +83 bp in the *APOA1* gene in the examined population (Table 3).

### 3.3. Alteration in the Activity of PON1, the Concentration of Lipid Profile Parameters and Selected Markers of Oxidative Stress in Terms of Genotypes for SNP rs662 in the PON1 Gene

A lower PON1(P) activity was found in the group of healthy subjects with the TT genotypes compared to the subjects with the TC genotype for SNP rs662 in the *PON1* gene (*p* = 0.0378). It was shown that PON1(P) and PON1(L) activities in the serum of AP patients were lower compared to healthy subjects in the case of individuals with the TT (*p* = 0.0047 and *p* = 0.0048, respectively) and TC genotypes (*p* < 0.0001 and *p* = 0.0111, respectively) for SNP rs662 in the *PON1* gene. These changes corresponded with lower HDL concentration and higher MDA concentration in the group of AP patients compared to healthy subjects with both examined genotypes for this SNP (TT genotypes: *p* = 0.0155 and *p* < 0.0001; TC genotype: *p* = 0.0275 and *p* = 0.0069, respectively). Additionally, a lower apoA-I concentration (*p* = 0.0125) was observed accompanied by a higher value of TC/HDL ratio (*p* = 0.0204) and LDL/HDL ratio (*p* = 0.0256) only in the case of individuals with the TT genotype for SNP rs662 in the *PON1* gene (Table 4).

A gradual decrease in PON1(A) and PON1(L) activities was shown during hospitalization of AP patients with the TT genotypes (*p* = 0.0331 and *p* = 0.0352 for comparison the first and seventh days). The value of the TC/HDL ratio increased during the hospitalization of AP patients with the TT genotype for SNP rs662 in the *PON1* gene (*p* = 0.0386 for comparison of the 1st and 3rd day). The changes were accompanied by increasing MDA concentration during the hospitalization of AP patients with the TT and TC genotypes for this SNP (*p* = 0.050 and *p* = 0.0476 for comparison of the 1st and 7th day, respectively) (Table 4).

The analyses of the results in terms of the exposure to tobacco smoke had shown a higher activity of PON1(A) and PON1(L) in the blood of smoking-healthy subjects with the TC genotype compared to the appropriate group of non-smokers. In the blood of smoking healthy subjects with the TT genotype, a lower HDL concentration and higher value of TC/HDL and LDL/HDL ratios compared to healthy non-smokers were shown, which were not associated with any changes in the PON1 activities (Table S1). However, in the group of smoking AP patients with the TT genotype, gradual decreases of the PON1(P), PON1(A), and PON1(L) activities were shown (*p* = 0.0458, *p* = 0.0376, and *p* = 0.0329, respectively, for comparison of the first and seventh day of hospitalization) in contrast to smoking AP patients with the TC genotype, where no changes in these parameters were found. These decreases in PON1 activities were not accompanied by changes in HDL and apoA-I concentrations (Figure S1).

### 3.4. Alteration in the Activity of PON1, the Concentration of Lipid Profile Parameters and Selected Markers of Oxidative Stress in Terms of Genotypes for SNP −75 bp in the APOA1 Gene

In the blood of AP patients with the GG genotype was shown a lower PON1(P) activity (more than 3-fold, *p* < 0.0001) and PON1(L) activity (near 2-fold, *p* = 0.0007) compared to healthy subjects with this genotype (Table 5). These changes were accompanied by lower PON1 concentration in the group of AP patients compared to healthy subjects with the GG genotype (*p* = 0.0019). Additionally, in the group of AP patients with this genotype, compared to appropriate group of healthy subjects, lower HDL (*p* = 0.0217) and apoA-I (*p* = 0.0026) concentrations were found, and a higher value of the LDL/HDL ratio (*p* = 0.0180) and MDA concentration (*p* < 0.0001) were found. Additionally, the blood of AP patients with the AA genotype showed a lower PON1(L) activity (*p* = 0.0384), lower HDL concentration (*p* = 0.0307), and higher MDA concentration (*p* = 0.0198) compared to healthy subjects with this genotype. A lower HDL concentration (*p* = 0.0104) accompanied by a higher MDA concentration (*p* = 0.0131) was also found in the blood of AP patients with the AG genotype compared to the appropriate group of healthy subjects (Table 5).

The differences in PON1 activities in terms of SNP −75 bp in the *APOA1* gene in the group of AP patients were found (Table 5). It was shown the highest activity of PON1(P) in the blood of AP patients with the AG genotype, which was statistically significant to AP patients with the GG (*p* = 0.0274 and *p* = 0.0123 on the third and seventh days of hospitalization, respectively) and AA genotypes (*p* = 0.0369 and *p* = 0.0475 on the third and seventh day of hospitalization, respectively). In the blood of AP patients with the AG genotype was also observed the highest PON1(L) activity, which was statistically significant to the AP patients with the GG (*p* = 0.0099 and *p* = 0.019 on the first and third days of hospitalization, respectively) and AA genotype (*p* = 0.0107 on the third day). The group of AP patients with the AA genotype was shown with the highest MDA concentration, which was statistically significant compared to patients with the GG and AG genotype (*p* = 0.0387 and *p* = 0.0069 on the first day, respectively). Additionally, during hospitalization of AP patients with the GG genotype showed an increasing MDA concentration (*p* = 0.0042 for comparison of the first and the seventh day) and the value of oxLDL/LDL ratio (*p* = 0.0457 for comparison the first and the third day and *p* = 0.0005 for comparison the third and seventh day) (Table 5).

The analyses of the results in terms of exposure to tobacco smoke had shown a lower HDL concentration in the smoking healthy subjects compared to healthy non-smokers in the case of all examined genotypes (Table S2). However, the concentration of apoA-I was lower only in the case of healthy smokers with the AA genotype when compared to healthy non-smokers. Interestingly, it was accompanied by higher activity of PON1(P) and PON1(A) in the blood of healthy smokers compared to non-smokers with this genotype. In the blood of smoking-healthy subjects with the AA genotype a higher value of LDL/HDL ratio, oxLDL/LDL ratio, and MDA concentration were shown compared to non-smokers. A higher value of the TC/HDL ratio in the group of smoking-healthy subjects with the AG genotype compared to non-smokers was also shown (Table S2).

The differences In parameters in terms of genotypes for examined SNP were also found. In the group of non-smoking healthy subjects with the AA genotype, the highest concentration of apoA-I (*p* = 0.0497 compared to non-smokers with the AG genotype) and HDL (*p* = 0.0498 compared to non-smokers with the GG genotype) was observed (Table S2). However, the group of healthy smokers with this genotype (AA) showed the highest value of TC/HDL ratio and LDL/HDL ratio, which was statistically significant when compared to the group of healthy smokers with the GG (*p* = 0.0229 and *p* = 0.0099, respectively) and AG genotype (*p* = 0.0259 and *p* = 0.0092, respectively) (Table S2).

The analyses of the results in terms of exposure to tobacco smoke have shown the highest concentration of apoA-I in the group of AP patients with the AG genotype, which was statistically significant compared to the individuals with the AA genotype (in non-smokers: *p* = 0.0041 on the first day; in smokers: *p* = 0.0003 on the seventh day) and the GG genotype (in non-smokers: *p* < 0.0001 and *p* = 0.0017 on the first and the seventh day, respectively) (Table S3). Additionally, the lowest apoA-I concentration was observed in the non-smoking individuals with the GG genotype, which was statistically significant compared to the subjects with the AA genotype on the first day (*p* = 0.0075). The differences in apoA-I concentrations between AP patients with the AA and GG genotypes were found in the smoking group (*p* = 0.0002 on the seventh day). Changes in the concentration of apoA-I according to genotype in the *APOA1* gene were associated with the changes in PON1 activities. The highest activity of PON1(P), PON1(A), and PON1(L) was noted in the group of AP patients with the AG genotype, which was statistically significant compared to appropriate individuals with the GG (in non-smokers—PON1(P): *p* = 0.0085, *p* = 0.0067 on the first and the third days; PON1(A): *p* = 0.0028 on the third day; PON1(L): *p* < 0.0001 and *p* = 0.0010 on the third and seventh days; in smokers—PON1(P): *p* = 0.0451 on the 7th day; PON1(A): *p* < 0.0001 and *p* = 0.0002 on the first and seventh days; PON1(L): *p* = 0.0081 and *p* = −0.0058 on the third and seventh days) and AA genotypes (in non-smokers—PON1(A): *p* = 0.0002 and *p* = 0.0208 on the third and the seventh days; in smokers—PON1(P): *p* = 0.0251 on the third day, PON1(A): *p* < 0.0001 on the first and seventh days; PON1 (L): *p* = 0.0004 and *p* < 0.0001 on the first and seventh days, respectively). Additionally, the lower PON1(L) activity in the non-smoking AP patients with the GG genotype compared to them with the AA genotype on the third day of hospitalization was found (*p* = 0.0020). In the group of smoking AP patients with the AA genotype, the lowest PON1 concentration was shown, which was statistically significant compared to those with the GG (*p* = 0.0031) and AG genotype (*p* = 0.0014) on the seventh day of hospitalization (Table S3).

The differences between smokers and non-smokers during the hospitalization of AP patients were shown. A higher concentration of apoA-I and PON1 in smokers with the GG genotype was found compared to the appropriate group of non-smokers (*p* = 0.0005 and *p* = 0.0310 on the 1st day, respectively). However, in the AP patients with the AG genotype, the apoA-I concentration was lower in smokers compared to non-smokers on the first and seventh days (*p* < 0.0001 and *p* = 0.0034, respectively), but this difference was inverted on the third day (*p* = 0.0048). Additionally, an increased PON1 concentration (*p* = 0.0077) accompanied by elevated PON1(A) activity (*p* < 0.0001) in smokers with the AG genotype compared to the adequate group of non-smokers on the first day was noted (Table S3).

The dynamic of the changes in examined parameters was shown as a gradual decrease in the PON1 concentration in smoking AP patients with the GG (*p* = 0.0234 for comparison the first day with the seventh day) and AG genotypes (*p* < 0.0001 for comparison on the first day with the third day and *p* = 0.0003 for comparison the first day with the seventh day). A decrease in the apoA-I concentration during hospitalization of non-smoking AP patients with the AG genotype was demonstrated (*p* = 0.0065 for comparison with the first day with the seventh day), where the lowest concentration of this parameter was noted on the third day of hospitalization, which was statistically significant compared to the first (*p* < 0.0001) and the seventh day (*p* = 0.0001). A decrease in the apoA-I concentration was also noted in the AP patients with the AA genotype (*p* = 0.0049 and *p* = 0.0116 for comparison on the first with the third day and first with the seventh day, respectively). A gradually decreasing HDL concentration during hospitalization of AP patients with the AA genotype was found, in both smoking (*p* = 0.0345 for comparison the first day with the seventh day) and non-smoking group (*p* < 0.0001 for comparison the first day with the third day) (Table S3).

### 3.5. Alteration in the Activity of PON1, the Concentration of Lipid Profile Parameters and Selected Markers of Oxidative Stress in Terms of Genotypes for SNP +83 bp in the APOA1 Gene

In the group of AP patients, in both individuals with the CC and TC genotypes, near 3-fold lower activity of PON1(P) (*p* < 0.0001 and *p* = 0.0252, respectively) accompanied by lower apoA-I (*p* = 0.0046 and *p* = 0.0432, respectively) and HDL concentrations (*p* = 0.0004 and *p* = 0.0058, respectively) compared with required groups of healthy subjects were shown (Table 6). These changes were associated with a higher value of TC/HDL ratio (*p* = 0.0052 and *p* = 0.0075, respectively) and MDA concentration (*p* < 0.0001 and *p* = 0.0005, respectively) in the groups of AP patients with above mentioned genotypes. A statistically significant increase in the value of LDL/HDL ratio in AP patients compared to healthy subjects was observed in the case of individuals with the TC genotypes (*p* = 0.0325). Additionally, only in the case of AP patients with the CC genotypes a lower PON1 concentration (*p* = 0.0056) and PON1(L) activity (*p* = 0.0424) was shown when compared to healthy subjects with the appropriate genotype (Table 6).

In the blood of AP patients with the CC genotypes a gradually decreasing concentration of apoA-I during hospitalization was shown (*p* < 0.0001 for comparison of the first and the seventh days), in contrast to the AP patients with the TC genotypes, where no change in the concentration of this parameter was observed. A decrease in apoA-I concentration in this group of AP patients was accompanied by gradually decreasing PON1 concentration (*p* = 0.0080 and *p* < 0.0001 for comparison on the first day with the third and the seventh days), which was statistically significant compared to AP patients with the TC genotype (*p* = 0.0045 on the first day and *p* = 0.0002 on the seventh day). Similarly, the activity of PON1(P) was decreased during the hospitalization of AP patients with the CC genotype (*p* = 0.0381 for comparison the first and seventh days), which was statistically significant compared to the AP patients with the TC genotype (*p* = 0.0237 and *p* < 0.0001 on the third and seventh days, respectively). A lower activity of PON1(A) was also observed in the AP patients with the CC genotype compared to individuals from this group with the TC genotype (*p* = 0.0101 and *p* = 0.0025 on the third and seventh days, respectively). It was found that a gradually decreasing PON1(L) activity during the hospitalization of AP patients with the CC genotype (*p* = 0.0105 and *p* = 0.0435 for comparison of the first with seventh day and the third with the seventh day, respectively), in contrast to the group of AP patients with the TC genotype (*p* = 0.0066 for comparison of AP patients with the CC and TC genotypes on the seventh day). Additionally, during hospitalization of AP patients with the CC genotype an increase in the value of LDL/HDL ratio (*p* = 0.0147 for comparison of the first and third days) was shown, and oxLDL/LDL ratio on the third day (*p* < 0.0001 for comparison of the first with the third day and the third with the seventh day) accompanied by gradually increasing MDA concentration (*p* = 0.0079 for comparison of the first with the seventh day) (Table 6).

In both investigated groups, healthy subjects and AP patients, no differences in the apoA-I and PON1 concentrations and its activities in the blood of smokers compared to non-smokers were shown (Table S4 and Figure S2). However, in the group of healthy subjects a lower HDL concentration was found in smokers compared to non-smokers in the individuals with all examined genotypes, corresponding with increased value of TC/HDL ratio and LDL/HDL ratio (Table S4).

Despite the fact that the dynamic of the changes in apoA-I and HDL concentrations no differences were shown between examined genotypes in both smokers and non-smokers (Figure S2), the concentration of PON1 was gradually decreased in smoking AP patients with the CC genotypes (*p* = 0.0266 for comparison of the first and seventh days). Additionally, a decrease in the concentration of this enzyme was observed in the blood of smoking AP patients with the TC genotypes on the third day of hospitalization (*p* = 0.0143 and *p* = 0.0426 for comparison of the first with the third day and the third with the seventh day, respectively) (Figure S2).

The decrease in the activities of PON1(P) and PON1(A) was found in the blood of AP patients with the CC genotypes compared to individuals with the TC genotypes (PON1(P): *p* = 0.0004 and *p* < 0.0001 on the third and seventh day, respectively in the group of non-smokers and *p* < 0.0001 on the seventh day in the group of smokers; PON1(A): *p* = 0.0009 and *p* = 0.0328 on the third and the seventh day, respectively, in the group of non-smokers). Additionally, a decrease in PON1(P) activity during hospitalization (*p* = 0.0441 was shown in the group of smoking AP patients with the CC genotype when compared to the first and the seventh days) (Figure S2).

### 3.6. The Results for the Odds Ratio Analysis and the Correlation Coefficients

The association between the occurrence of AP and male sex was shown. In the group of men with the TT genotype for SNP rs662 in the *PON1* gene, the risk of AP occurrence was more than three-fold higher compared to women (OR = 3.2500, *p* = 0.0083). In the case of men with the TC genotype for this SNP, this association was not statistically significant (OR = 6.0000, *p* = 0.2053). The risk of AP recurrence was shown to be higher (more than five-fold) in the group of men with *the* GG genotype for −75 bp (OR = 5,6220, *p* = 0.0070) and the CC genotype for SNP +83 bp (OR = 5.1019, *p* = 0.0026) in the *APOA1* gene compared to women. In the case of male individuals with the AA and AG genotype for −75 bp and the TC genotype for SNP +83 bp in the *APOA1* gene, this association was not statistically significant (respectively: OR = 2,6667, *p* = 0.4870; OR = 2,4059, *p* = 0.3687; OR = 2,2500, *p* = 0.5478). The association of haplotype for examined SNPs with acute pancreatitis was analyzed. The results of this analysis in Table S5 were presented. Polymorphisms (SNP rs662 in the *PON1* gene, SNP rs670, and rs5069 in the APOA1 gene) were also analysed for linkage disequilibrium (Table S6).

Statistically significant correlations in terms of examined genotypes for SNP rs622 in the *PON1* gene and SNP −76 bp and +83 bp in the *APOA1* gene were presented in Table 7.

## 4. Discussion

There is evidence of the role of PON1 in the anti-inflammatory and antioxidant properties ascribed to HDL [16,20]. Apolipoprotein A-I is one of the major structural and functional protein constituents of HDL [11,21]. The activity of PON1 can be influenced by changes in the apoA-I-HDL complex into which the PON1 molecule is incorporated [22,23]. The current study was undertaken to investigate the polymorphic sites in the *PON1* gene (rs662) and *APOA1* gene (rs670 and rs5069) in individuals suffering from acute pancreatitis, from which data was hitherto not available.

There are reports showing that the T allele for SNP rs662 in the *PON1* gene is related to the decreased PON1 activities in serum compared to C alleles, which predispose to higher hydrolytic activity of this enzyme [5,7,24]. This is in accordance with our results, in which a decrease in PON1(P) activity in healthy individuals with TT genotype compared to TC genotype was shown. In the study conducted by Scherrer et al. [25] it was shown that the genotype for the SNP rs662 has an impact on the hydrolytic activity of PON1 toward the substrate paraoxone. Despite the fact that the PON(P) activity in the blood AP patients with the TT genotype was not changed in comparison to the patients with the TC genotype, a decrease was shown in the activity of PON1(A) and PON1(L) during hospitalization of AP patients with the TT genotype, which can confirm the influence of this genotype on the decreased activity of PON1. PON1 is recognized as an enzyme with antioxidative and anti-inflammatory potential [26,27]. So, a decrease in PON1 activity accompanied with the intensification of inflammation may be an important inflammatory indicator [28]. It can explain an increased risk of AP occurrence in individuals with the TT genotype, which was shown in this study. The diversified impact of the SNP rs662 on specific activities of PON1 may result from the fact that this polymorphism is based on the amino acid substitution, which contributes to a change in the conformation of the PON1 molecule, and thus changes its affinity for binding various substrates [29]. An additional factor changing the affinity of the enzyme for binding various substrates may be oxidative stress induced by inflammation [30]. The influence of the TT genotype for SNP rs662 in the *PON1* gene on the inhibition of PON1 activities was especially shown in the group of smoking AP patients. In this group, in contrast to smoking AP patients with the TC genotype, a gradual decrease of PON1(P), PON1(A), and PON1(L) activities during hospitalization was found. Moreover, these changes were not associated with the decreased concentration of HDL and apoA-I. It can indicate that the TT genotype contributes directly to lower activities of PON1 in AP patients, but tobacco smoke exposure can be recognized as an additional factor influencing PON1 activities, which is consistent with other studies [31].

In our study, two SNPs (rs670 and rs5069) in the *APOA1* gene playing an important role in lipid metabolism were analyzed [32,33]. It has been found that the SNP rs670 in the *APOA1* gene (−75 G/A) was associated with gallstone disease, as a major risk factor for AP [34]. Our previous studies have shown that the course of AP is related to lipids metabolism disturbances, which have reflected decreased HDL concentrations and increased levels of lipids peroxidation products in the blood [16]. In this study, the analysis of the results according to SNP rs670 in the *APOA1* gene confirmed these findings in the group of AP patients with all genotypes. Moreover, it had shown that these disturbances are especially prominent in AP patients with the GG genotype, which was additionally associated with the decreased PON1 and apoA-I concentrations accompanied by decreased PON1(P) and PON1(L) activities in the blood of AP patients compared to healthy subjects. Therefore, in the non-smoking AP patients with this genotype was observed the lowest apoA-I concentration accompanied by decreased PON1(L) activity, which was statistically significant to the AP patients with the AA genotype. ApoA-I binds to the PON1 molecule and stabilizes this enzyme [23]. It was known that the binding of apoA-I to PON1 selectively stimulates PON1(L) activity [23]. ApoA-I is not necessary for the binding of PON1 on HDL; however, it is important for the stability and activity of enzymes [35]. So, a decrease in apoA-I concentration disturbing the PON1(L) activity against oxidized polyunsaturated fatty acids on lipoproteins can result in increased oxidative modification of LDL [36]. In our study, this process was reflected in the increased oxLDL/LDL ratio in the group of AP patients with the GG genotype on the third day of hospitalization compared to the first day. It can indicate intensive oxidative stress associated with inflammation. It may explain the increased risk of AP occurrence in the individuals with the GG genotype for SNP rs670 in the *APOA1* gene shown in this study.

In comparison to other examined genotypes for SNP rs670, our results have shown the highest concentration of apoA-I in the blood of non-smoking healthy subjects with the AA genotype associated with the highest HDL concentration. However, simultaneous consideration of the SNP rs670 and the exposure to tobacco smoke xenobiotic in the course of AP has shown the highest apoA-I concentration in the blood of AP patients with the AG genotype in comparison to other genotypes, which was related to the increased PON1 activities (in both, smokers and non-smokers). These results are in accordance with the other reports describing a direct effect on the A allele for SNP rs670 in the *APOA1* gene on the transcription of the *APOA1* gene that correlates with a higher blood concentration of this apolipoprotein [37,38]. The exact mechanism of this association is still unclear [37]. However, the results have been inconsistent and inconclusive, with few studies reporting either no relationship or a negative association between APOA1 −75 A allele and plasma lipids [32,39].

Our study showed that increased apoA-I concentration in the blood of AP patients with the A allele for SNP rs670 is gradually decreased during the hospitalization of these individuals, which was not accompanied by the changes in PON1 concentration and its activities. These changes were statistically significant in the non-smoking group with the AG and AA genotypes. It can indicate that inflammation can contribute to decreased apoA-I concentration, which was reported earlier [40]. It was shown that inflammatory cytokines, such as TNF and IL-1β, repress the production of apoA-I in hepatocytes [12]. Moreover, in the AP patients with the A allele for SNP rs670 (the AA and AG genotypes) a prominent influence of tobacco smoke exposure on the decrease in apoA-I concentration was observed. This change was visible in the group of smoking-healthy subjects with the AA genotype, which corresponded with the elevated lipids peroxidation manifesting in increased oxLDL/LDL ratio and MDA concentration. A negative correlation between the concentrations of apoA-I and MDA showed in the group of AP patients with the AA genotype can also confirm the association of decreased apoA-I level with intensified lipid peroxidation. Interestingly, these changes were accompanied by an increase in PON1(P) and PON1(A) activities. These results can confirm the finding of Meisinger et al. [41] which revealed that increasing levels of inflammatory cytokines (e.g., associated with tobacco smoking) cause increased arylesterase and paraoxonase activities. On the other hand, this research also found that high levels of inflammation may lead to impaired enzyme activity and subsequently to dysfunctional HDL [41]. These findings can be confirmed by the analysis of the dynamic of the changes in above mentioned parameters in the group of AP patients conducted in our study. In the group of smoking AP patients with the AG genotype, a decrease in apoA-I concentration on the first and seventh days of hospitalization corresponded with increased PON1 concentration on admission (in comparison to the appropriate group of non-smokers), what was decreased on the seventh day. However, a decreased PON1 concentration in AP patients with the AG genotype was not associated with gradually decreasing PON1 activities during seven days of hospitalization. Insufficient observation time to find the changes in PON1 activities may have resulted in the decreased concentration of this protein. The changes observed in the AP patients with the AA genotype can also confirm the sensitivity of the individuals with A allele to the changes in PON1 concentration. In these subjects, a gradually decreased HDL concentration during hospitalization was shown, in both smokers and non-smokers, but the lowest PON1 concentration was found only in the smoking group of AP patients with the AA genotype on the seventh day of hospitalization, which was statistically significant compared to the GG and AG genotype. It can indicate that smoking and inflammation in the subjects with A allele can influence on PON1 synthesis. In the study conducted by Kahraman et al. [42], the effect of tobacco smoke on decrease in PON1 was found. Moreover, it is reported that the proinflammatory cytokines, such as IL-1β and TNFα, downregulate PON1 expression and secretion by liver cells via NF-κB [27].

Proinflammatory cytokines can also indirectly repress the production of apoA-I from hepatocytes [27]. This effect is associated with the influence of serum amyloid A (SAA) on HDL metabolism. SSA is a major acute phase reactant whose secretion from the liver is significantly increased during inflammation [9,12,43]. The presence of SAA can disturb apoA-I-lipidation, a key event in HDL biogenesis, modulating the interaction between apoA-I molecule and lipid transporter ABCA1 or impairs ABCA1-dependent efflux of cholesterol from macrophages [12]. This process causes perturbation of HDL core and surface lipid leading to the generation of small, lipid-depleted apoA-I that is susceptible to catabolism by scavenger receptor class B type I (SR-BI) [43]. Consequently, lipid-poor apoA-I is rapidly catabolized in the liver and the kidney [27]. It was reported that a reduction in nascent HDL formation may be partly responsible for reduced HDL concentration during inflammation [43]. It was evidenced that inflammation accompanied by prolonged SAA secretion by the liver is associated with lower apoA-I and PON1 activity, which reflects the intensification of lipid peroxidation [27].

Our study has shown that the above-described process can be pronounced in the course of AP. It can be confirmed by decreased concentrations of apoA-I and HDL resulting in decreased PON1(P) activity in the blood of AP patients compared to healthy subjects in both individuals with the CC and TC genotype for SNP rs5069 in the *APOA1* gene. Huang et al. [44] also observed a reduced serum level of the apoA-I in the inflammatory state. Other authors were shown the association of decreased apoA-I level and HDL concentration with lower PON1 activity [23]. Moreover, our study found that the CC genotype for the above-mentioned SNP can deepen these changes, which confirmed a gradually decreasing concentration of apoA-I during the hospitalization of AP patients with this genotype, associated with lower PON1 concentration and its all activities, which was statistically significant compared to AP patients with the TC genotype. These disturbances, especially gradually decreasing PON(L) activity during hospitalization AP patients with the CC genotype, resulted in the intensification of lipids peroxidation manifested in increasing the value of the oxLDL/LDL ratio and the MDA concentration. In another study, it was shown that apoA-I plays a protective role in inflammatory reactions [44]. So, disturbances in the synthesis of this apolipoprotein inducing the changes in PON1 activity can be related to the increased (more than five-fold) risk of AP occurrence in individuals with the CC genotype compared to those with the TC genotype. Additionally, our study confirmed that tobacco smoking can cause unfavorable changes in lipid profile and a decrease in PON1 concentration independent of the genotype for SNP rs5069 in the *APOA1* gene. Attention was paid to the fact that despite the lack of the differences between smokers and non-smokers in apoA-I concentration, the activity of PON1(P) was gradually decreasing during hospitalization of smoking AP patients with the CC genotype, in contrast to the adequate group of non-smokers. It can indicate that oxidative stress associated with inflammation and the exposure to smoke xenobiotics may contribute to a decrease in PON1(P) activity in the individuals with the CC genotype. It confirms the particular susceptibility of this genotype to changes in pro/antioxidative balance.

The information about the influence of SNP rs5069 on apoA-I concentration and lipid profile parameters is contradictory. Chen et al. [45] have not shown any relationship between the concentration of lipid profile parameters and SNP rs5069. Feng et al. [14] also found no association of the above-mentioned SNP with lipid metabolism. However, in other studies [15,33] increased concentrations of apoA-I and HDL were found in the subjects with the C allele for this SNP. However, in our study, the influence of the CC genotype for SNP rs5069 in the *APOA1* gene on a decreased blood apoA-I concentration was shown. Possibly due to differences in the sample groups between the studies (ethnicity, sex, etc.) our findings are not consistent with those reported in other research. The other reason can be the fact that there is no research on the topic under consideration in acute pancreatitis. These reasons make the comparison of our results to earlier studies is difficult. Our results are limited due to the sample size and they have to be confirmed in the future.

The mechanisms by which the apoA-I and PON1 influence HDL metabolism are probably complex. Moreover, full knowledge and understanding of the influence of these two molecules on HDL concentration hinder the possibility of the occurrence of novel, as yet unknown mutations. Additionally, lifestyle factors, including cigarette smoking, increase the mutation burden. Unknowns mutations may interact in a hitherto unknown way with the studied genetic changes and have a serious impact on HDL metabolism, leading to apoA-I and HDL deficiencies resulting in clinical complications.

## 5. Summary

**Figure 2:**
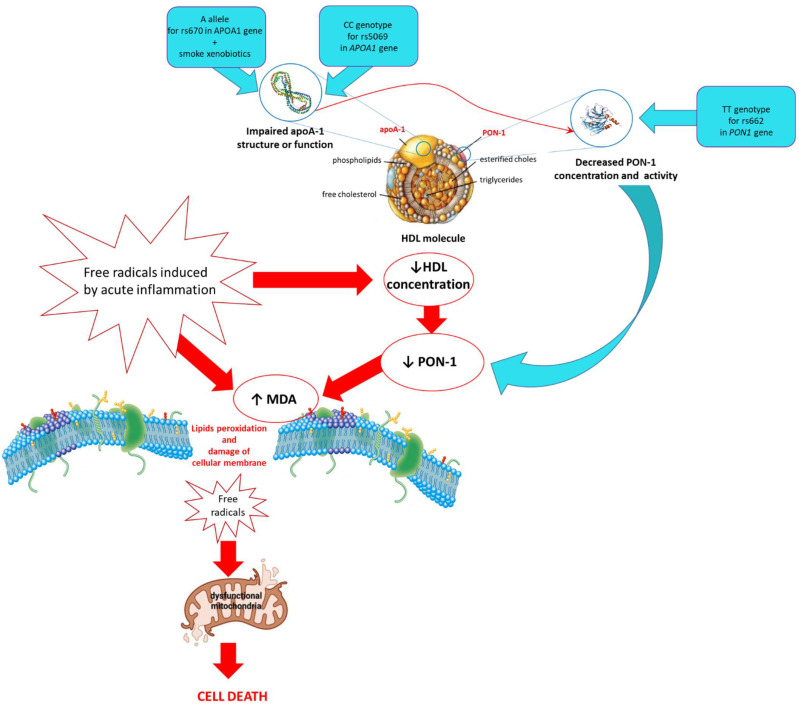
The involvement of environmental and genetic factors (SNPs: rs662, rs670 and rs5069) in the disorder of cellular pro/antioxidant balance in acute pancreatitis.

In the course of AP was observed an intensified process of lipid peroxidation, which was manifested in the increased concentration of MDA in the blood of patients compared to healthy individuals. In these patients, a decreased HDL concentration as a preventing factor against cell membrane oxidation was also shown. The changes in HDL concentration or oxidative modifications affect PON1 and apoA-I molecule located on its surface, which has a decisive influence on the antioxidant status. These parameters varied depending on genetic (SNPs) and environmental factors (exposure to tobacco smoke). It was shown that oxidative stress associated with exposure to tobacco smoke and inflammation in the subjects with A allele for SNP rs670 in the *APOA1* gene could contribute to reduced PON1 synthesis. However, the occurrence of this allele was related to the highest concentration of apoA-I in the blood of non-smoking healthy subjects accompanied by the highest HDL concentration in comparison to other genotypes. A decreased apoA-I concentration in the blood of individuals with the CC genotype for SNP rs5069 in the *APOA1* gene was the likely cause of the reduction of PON1 concentration and its activities, which resulted in the intensification of lipids peroxidation. Moreover, the TT genotype for SNP rs662 in the *PON1* gene is predisposed to lower activities of PON1 (Figure 2). Additionally, the mathematical model used to assess the risk of disease occurrence showed that the individuals with the TT genotype for rs662 in the *PON1* gene, GG genotype for SNP rs670, and CC genotype for SNP rs5069 in the *APOA1* gene have an increased chance of developing AP. Our results indicate that both, environmental and genetic factors can change the antioxidative potential of the pancreas to neutralize oxidative stress.

## Figures and Tables

**Table 1:** Clinical characteristics of the healthy subjects and patients with acute pancreatitis on the first day of hospitalization.

Parameters	Mean ± SD	Mean ± SD	
**Healthy Subjects**			
	**Non-Smokers (*n* = 23)**	**Smokers (*n* = 28)**	***p***
Gender [% female/% male]	83%/17%	54%/46%	-
Age [years]	45.7 ± 8.7	46.1 ± 8.4	0.8684
BMI [kg/m^2^]	22.7 ± 1.6	23.0 ± 2.1	0.5757
hsCRP [mg/L]	0.6 ± 0.2	0.4 ± 0.1	<0.0001
**The patients with acute pancreatitis**			
	**Non-smokers (*n* = 17)**	**Smokers (*n* = 27)**	***p***
Gender [% female/% male]	53%/47%	26%/74%	-
Age [years]	47.3 ± 15.0	48.2 ± 13.3	0.8979
BMI [kg/m^2^]	27.6 ± 5.9	24.1 ± 4.3	0.1411
hsCRP [mg/L]	112.3 ± 128.3	101.9 ± 99.2	0.8318
Lipase [U/L]	120.7 ± 98.9	213.5 ± 177.0	0.2726
Amylase [U/L]	529.1 ± 692.0	449.8 ± 732.1	0.7601
ALT [U/L]	27.1 ± 20.2	30.2 ± 20.6	0.7145
AST [U/L]	102.2 ± 173.1	71.5 ± 77.3	0.5648
GGT [U/L]	227.0 ± 275.0	273.6 ± 295.6	0.7087
Erythrocytes [10^12^/L]	4.2 ± 1.0	4.4 ± 0.9	0.6680
Leukocytes [10^9^/L]	13.1 ± 3.4	12.3 ± 4.0	0.6379
Hemoglobin [g/dL]	12.6 ± 3.1	13.5 ± 2.5	0.4443
Hematocrit [%]	36.9 ± 7.7	39.8 ± 6.3	0.3498
Bilirubin (total) [mg/dL]	1.7 ± 1.6	1.8 ± 2.2	0.8383
Alkaline phosphatase [U/L]	250.2 ± 317.9	157.3 ± 119.7	0.3276
Urea [mg/dL]	35.7 ± 34.7	31.3 ± 27.2	0.7420
Creatinine [mg/dL]	1.6 ± 1.7	2.4 ± 5.4	0.7076
eGFR [mL/min/1.73 m^2^]	67.9 ± 30.5	91.4 ± 42.3	0.2010
Total protein [g/dL]	6.5 ± 0.5	6.4 ± 0.6	0.9578
Total cholesterol [mg/dL]	158.0 ± 38.2	163.0 ± 24.6	0.9322
Sodium [mmol/L]	136.6 ± 3.5	129.7 ± 28.6	0.5088
Potassium [mmol/L]	4.0 ± 0.5	4.1 ± 0.6	0.7125
Cotinine [ng/mL]	6.8 ± 14.6	128.8 ± 58.6	<0.0001

Values shown as mean and SD.

**Table 2:** The activities of PON1, its concentration, the concentration of apoA-I, lipid profile parameters, and selected markers of pro/antioxidative balance in the context of intersexual variability in the group of healthy subjects and AP patients.

Parameters	Healthy Subjects		*p*	The Patients with Acute Pancreatitis		*p*
	Women (*n* = 34)	Men (*n* = 17)		Women (*n* = 16)	Men (*n* = 28)	
**PON1**(**P**) **activity** **[U/L]**	265.9 ± 139.4 (147.2; 225.4; 395.9)	228.7 ± 170.2 (150.6; 179.7; 245.7)	0.4112	68.5 ± 44.1 ** (42.2; 62.5; 79.8)	85.6 ± 48.8 *** (48.9; 79.0; 48.8)	0.2871
PON1(A) **activity** **[U/L]**	115.0 ± 42.2 (85.8; 113.3; 136.3)	109.5 ± 48.0 (80.7; 115.3; 145.9)	0.6760	136.7 ± 55.6 (93.2; 142.0; 175.3)	124.6 ± 63.5 (84.2; 126.9; 63.5)	0.5392
**PON1**(**L**) **activity** **[U/L]**	8.5 ± 3.0 (6.3; 8.5; 10.7)	8.2 ± 4.0 (5.8; 8.6; 10.1)	0.7727	6.3 ± 2.1 ** (5.0; 5.7; 8.7)	6.3 ± 3.2 (4.2; 5.9; 3.2)	0.9694
**PON1 concentration [µg/mL]**	25.2 ± 7.3 (21.0; 26.3; 30.0)	21.5 ± 5.9 (17.5; 20.7; 26.8)	0.1413	19.1 ± 8.7 (14.6; 16.1; 25.1)	19.8 ± 11.2 (14.7; 16.9; 11.2)	0.8657
**ApoA-I concentration ** **[g/L]**	1.4 ± 0.4 (1.0; 1.3; 1.7)	1.5 ± 0.4 (1.2; 1.4; 1.6)	0.3788	1.1 ± 0.6 (0.6; 1.0; 1.3)	0.8 ± 0.5 *** (0.4; 0.7; 0.5)	0.2893
**HDL concentration** **[mg/dL]**	68.8 ± 14.6 (57.5; 69.5; 80.0)	57.2 ± 11.3 * (49.0; 56.0; 65.0)	**0.0066**	50.9 ± 21.2 ** (31.6; 52.0; 67.0)	45.5 ± 19.4 *** (29.1; 44.7; 19.4)	0.4267
**TC/HDL ratio**	3.2 ± 1.1 (2.5; 2.8; 3.5)	3.4 ± 0.5 (3.0; 3.4; 3.8)	0.3359	4.0 ± 2.0 (2.5; 2.7; 6.1)	4.3 ± 2.3 (2.6; 3.9; 2.3)	0.7647
**LDL/HDL ratio**	1.8 ± 0.9 (1.3; 1.6; 2.0)	2.1 ± 0.5 (1.8; 2.1; 2.4)	0.3388	2.3 ± 1.7 (1.2; 1.4; 3.8)	2.7 ± 2.1 (1.1; 2.3; 2.1)	0.5568
**oxLDL/LDL ratio** **[U/g]**	54.5 ± 22.2 (39.9; 49.5; 63.7)	53.1 ± 28.8 (33.2; 54.0; 61.0)	0.8743	71.8 ± 39.1 (50.4; 60.5; 83.2)	60.2 ± 46.4 (27.9; 36.9; 46.4)	0.5991

Values shown as mean and SD (first quartile, median, third quartile). * *p* < 0.05 compared to women, ** *p* < 0.05 compared to healthy women, *** *p* < 0.05 compared to healthy men.

**Table 3:** The frequency of genotypes for SNP rs662 in the *PON1* gene and SNP −75 bp and +83 bp in the *APOA1* gene in the group of patients with acute pancreatitis and healthy subjects.

Polymorphism	Genotype	Healthy Subjects	AP Patients	OR (95% CI)	*p*-Value
**rs662** **(*PON1*)**	T/T	44 (86.3%)	40 (90.9%)	1.00	0.48
	T/C	7 (13.7%)	4 (9.1%)	1.59 (0.43–5.84)	
**rs670 (−75 G/A)** **(*APOA1*)**	G/G	32 (62.8%)	23 (52.3%)	1.00	0.036
	A/G	15 (29.4%)	9 (20.4%)	1.20 (0.45–3.21)	
	A/A	4 (7.8%)	12 (27.3%)	0.24 (0.07–0.84)	
	A/G	15	9	1.00	0.23
	G/G–A/A	36	35	0.56 (0.22–1.44)	
**rs5069 (+83 C/T)** **(*APOA1*)**	C/C	44 (86.3%)	29 (65.9%)	1.00	0.018
	T/C	7 (13.7%)	15 (34.1%)	0.31 (0.11–0.85)	

**Table 4:** The activities of PON1, its concentration, the concentration of apoA-I, lipid profile parameters, and selected markers of pro/antioxidative balance in the context of genotypic variability of rs662 in the *PON1* gene in the group of healthy subjects and AP patients.

Parameters	Genotypes	Healthy Subjects (*n* = 51)	The Patients with Acute Pancreatitis (*n* = 44)		
			1st Day of Hospitalization	3rd Day of Hospitalization	7th Day of Hospitalization
**PON1**(**P**) **activity** **[U/L]**	T/T	214.0 ± 114.3	80.7 ± 49.1 *	85.7 ± 74.5	66.2 ± 67.7
		(144.7; 173.4; 264.9)	(43.4; 71.2; 99.1)	(32.2; 52.0; 137.1)	(24.4; 49.7; 92.8)
	T/C	341.8 ± 160.7 **	67.0 ± 28.0 *	73.5 ± 54.1	81.1 ± 64.0
		(229.8; 288.9; 472.7)	(44.2; 61.7; 89.8)	(34.6; 62.2; 112.4)	(38.2; 50.4; 154.7)
PON1(A) **activity** **[U/L]**	T/T	111.9 ± 45.5	128.9 ± 63.5	106.0 ± 55.9	93.5 ± 54.5 #
		(81.7; 115.3; 142.8)	(87.6; 135.4; 165.3)	(53.6; 109.2; 143.8)	(49.6; 94.0; 138.3)
	T/C	121.3 ± 33.1	124.4 ± 37.2	107.1 ± 30.6	107.9 ± 46.2
		(100.3; 112.7; 145.9)	(96.1; 115.2; 152.8)	(82.3; 103.4; 131.9)	(75.9; 86.9; 160.9)
**PON1**(**L**) **activity** **[U/L]**	T/T	8.5 ± 3.4	6.4 ± 2.9 *	5.6 ± 2.6	4.9 ± 2.2 #
		(6.1; 8.7; 10.6)	(4.6; 5.8; 8.7)	(3.9; 5.6; 6.9)	(3.2; 4.1; 6.7)
	T/C	7.7 ± 3.1	4.7 ± 2.0 *	4.2 ± 1.7	3.8 ± 1.9
		(5.5; 6.4; 10.1)	(3.1; 4.3; 6.3)	(2.8; 4.3; 5.6)	(2.6; 2.7; 6.0)
**PON1 concentration [µg/mL]**	T/T	23.4 ± 7.1	20.3 ± 10.4	16.6 ± 8.2	14.5 ± 7.4
		(17.8; 24.6; 28.3)	(14.7; 16.4; 29.2)	(10.5; 13.9; 21.7)	(8.6; 13.5; 21.2)
	T/C	25.3 ± 6.6	16.2 ± 6.4	10.4 ± 2.7	12.0 ± 7.2
		(21.6; 25.8; 29.7)	(11.8; 16.8; 20.5)	(8.5; 10.3; 12.3)	(7.2; 8.6; 20.3)
**ApoA-I concentration ** **[g/L]**	T/T	1.4 ± 0.4	0.9 ± 0.5 *	0.8 ± 0.6	0.7 ± 0.3
		(1.0; 1.3; 1.6)	(0.6; 0.7; 1.2)	(0.3; 0.4; 1.3)	(0.5; 0.7; 0.7)
	T/C	1.6 ± 0.4	1.0 ± 0.8	0.6 ± 0.4	0.3 ± 0.1
		(1.4; 1.6; 1.8)	(0.4; 0.7; 1.6)	(0.3; 0.5; 0.8)	(0.1; 0.4; 0.4)
**HDL concentration [mg/dL]**	T/T	64.1 ± 15.1	54.0 ± 31.9 *	38.8 ± 25.8	44.9 ± 24.9
		(52.0; 63.0; 74.0)	(29.7; 47.1; 67.6)	(17.8; 32.1; 56.4)	(25.0; 42.2; 62.8)
	T/C	68.7 ± 10.5	46.0 ± 18.7 *	49.9 ± 24.2	48.3 ± 41.0
		(59.0; 69.0; 81.0)	(31.5; 46.7; 60.5)	(29.7; 46.5; 70.1)	(21.3; 28.0; 95.6)
**TC/HDL ratio**	T/T	3.3 ± 1.0	4.5 ± 3.2 *	6.4 ± 5.8 #	5.2 ± 2.9
		(2.7; 3.0; 3.7)	(2.5; 3.7; 6.1)	(2.6; 4.5; 8.8)	(2.8; 5.0; 6.8)
	T/C	2.9 ± 0.6	8.0 ± 6.7	5.1 ± 3.2	4.0 ± 3.7
		(2.5; 2.7; 3.7)	(4.3; 5.3; 11.7)	(2.6; 4.7; 7.7)	(1.4; 4.0; 6.6)
**LDL/HDL ratio**	T/T	1.9 ± 0.8	3.0 ± 3.1 *	4.4 ± 5.0	3.5 ± 2.2
		(1.4; 1.8; 3.4)	(1.1; 2.2; 4.1)	(1.2; 3.1; 5.8)	(1.6; 3.6; 4.4)
	T/C	1.7 ± 0.5	6.5 ± 6.6	3.5 ± 2.5	6.8 ± 10.8
		(1.2; 1.6; 2.3)	(2.9; 3.8; 10.1)	(1.8; 2.7; 5.3)	(0.0; 1.2; 19.3)
**oxLDL/LDL ratio** **[U/g]**	T/T	55.8 ± 26.1	67.6 ± 38.8	89.9 ± 36.9	63.7 ± 30.6
		(40.4; 53.9; 60.9)	(39.0; 54.2; 86.5)	(69.7; 92.3; 117.8)	(43.0; 68.3; 90.6)
	T/C	44.8 ± 16.5	66.8 ± 53.0	90.2 ± 47.2	84.6 ± 36.4
		(31.8; 41.1; 63.2)	(30.4; 51.6; 103.3)	(42.1; 92.1; 136.5)	(46.7; 80.4; 126.3)
**MDA concentration [nmol/µL]**	T/T	0.8 ± 0.6	2.2 ± 0.5 *	2.6 ± 0.7	2.9 ± 0.9 #
		(0.3; 0.6; 1.1)	(1.9; 2.2; 2.5)	(2.2; 2.5; 2.9)	(2.3; 2.9; 3.4)
	T/C	1.2 ± 0.8	2.4 ± 0.6 *	2.8 ± 0.4	3.4 ± 0.2 #
		(0.3; 1.1; 2.2)	(2.0; 2.4; 2.8)	(2.4; 2.8; 3.1)	(3.2; 3.3; 3.6)

Values shown as mean and SD (1st quartile, median, 3rd quartile). * *p* < 0.05 compared to healthy subjects with the same genotype, ** *p* < 0.05 compared to subjects with the TT genotype, ^#^ *p* < 0.05 compared to the 1st day.

**Table 5:** PON1 activities, its concentration, the concentration of apoA-I, lipid profile, and selected markers of pro/antioxidative balance in the context of genotypic variability of SNP −75 bp in the *APOA1* gene in the group of healthy subjects and AP patients.

Parameters	Genotypes	Healthy Subjects (*n* = 51)	The Patients with Acute Pancreatitis (*n* = 44)		
			1st Day of Hospitalization	3rd Day of Hospitalization	7th Day of Hospitalization
**PON1**(**P**) **activity** **[U/L]**	G/G	261.1 ± 158.5	69.2 ± 43.2 *	67.5 ± 59.4	44.9 ± 35.5
		(145.1; 179.7; 389.1)	(42.8; 61.3; 78.8)	(32.3; 48.3; 85.4)	(26.3; 33.3; 52.1)
	A/A	299.5 ± 252.5	61.2 ± 37.2	52.1 ± 32.7	40.8 ± 22.7
		(144.2; 163.1; 591.1)	(42.8; 47.2; 89.0)	(31.7; 46.9; 53.2)	(19.4; 38.2; 64.7)
	A/G	229.3 ± 116.0	123.1 ± 30.3	173.6 ± 39.2 **/***	127.2 ± 38.9 **/***
		(151.6; 183.3; 267.3)	(101.7; 123.1; 144.5)	(147.3; 155.0; 218.7)	(99.7; 127.2; 154.7)
PON1(A) **activity** **[U/L]**	G/G	108.0 ± 47.1	105.9 ± 49.3	97.3 ± 52.0	92.1 ± 53.5
		(81.7; 108.2; 139.8)	(61.1; 117.5; 141.7)	(46.2; 104.4; 150.4)	(49.6; 75.9; 138.3)
	A/A	137.6 ± 13.1	104.5 ± 75.5	100.6 ± 50.8	63.8 ± 41.3
		(122.6; 144.5; 145.9)	(28.9; 93.2; 134.5)	(51.2; 98.2; 119.8)	(29.3; 61.5; 98.4)
	A/G	119.0 ± 39.6	137.1 ± 49.6	140.3 ± 3.2	138.6 ± 31.6
		(90.0; 115.3; 150.6)	(80.8; 156.2; 174.2)	(138.1; 138.8; 144.0)	(116.2; 138.6; 160.9)
**PON1(L) activity** **[U/L]**	G/G	7.9 ± 3.1	4.5 ± 2.1 *	4.2 ± 1.7	3.7 ± 1.9
		(5.8; 8.1; 9.6)	(3.8; 4.5; 5.3)	(3.0; 4.1; 5.7)	(2.5; 3.2; 5.5)
	A/A	10.1 ± 0.7	6.0 ± 3.4 *	5.4 ± 2.9	4.7 ± 2.8
		(9.4; 10.0; 10.8)	(2.4; 5.4; 7.7)	(2.4; 5.4; 7.7)	(2.9; 3.6; 6.5)
	A/G	9.1 ± 3.9	9.1 ± 3.5 ***	9.3 ± 3.6 **/***	6.6 ± 0.8
		(6.4; 8.6; 11.0)	(6.8; 7.4; 13.1)	(5.8; 9.3; 12.9)	(6.0; 6.6; 7.1)
**PON1 concentration [µg/mL]**	G/G	25.9 ± 6.1	15.6 ± 5.5 *	12.9 ± 5.0	11.5 ± 6.4
		(20.6; 26.3; 30.1)	(14.7; 15.9; 17.3)	(9.8; 12.1; 18.5)	(3.6; 8.6; 22.2)
	A/A	28.7 ± 0.4	20.1 ± 13.7	10.4 ± 3.3	15.9 ± 5.8
		(28.4; 28.7; 29.0)	(7.8; 17.7; 34.8)	(7.1; 10.4; 13.7)	(12.1; 15.3; 18.7)
	A/G	18.7 ± 6.6	23.3 ± 12.8	15.6 ± 8.9	11.6 ± 6.3
		(15.8; 19.3; 23.8)	(13.2; 23.1; 29.3)	(10.2; 14.8; 22.8)	(7.8; 11.2; 15.1)
**ApoA-I concentration** **[g/L]**	G/G	1.5 ± 0.4	0.8 ± 0.5 *	0.9 ± 0.6	0.6 ± 0.2
		(1.2; 1.4; 1.7)	(0.5; 0.9; 1.3)	(0.5; 0.9; 1.3)	(0.4; 0.6; 0.7)
	A/A	1.5 ± 0.6	1.0 ± 0.7	0.5 ± 0.4	0.4 ± 0.3
		(1.0; 1.5; 2.1)	(0.6; 0.7; 1.6)	(0.3; 0.4; 0.8)	(0.2; 0.4; 0.6)
	A/G	1.2 ±0.3	1.0 ± 0.8	0.9 ± 0.9	0.8 ± 0.6
		(1.0; 1.3; 1.4)	(0.4; 0.7; 1.9)	(0.4; 0.5; 1.9)	(0.4; 0.8; 1.2)
**HDL concentration [mg/dL]**	G/G	62.7 ± 12.8	50.3 ± 21.9 *	47.9 ± 28.3	55.9 ± 24.3
		(53.0; 63.0; 72.0)	(28.3; 52.8; 70.0)	(21.5; 41.2; 74.9)	(39.7; 47.6; 73.9)
	A/A	67.3 ± 23.6	41.2 ± 15.1 *	30.1 ± 25.1	30.1 ± 19.5
		(49.0; 59.0; 94.0)	(29.2; 41.5; 44.6)	(14.1; 23.6; 32.1)	(16.5; 21.3; 46.3)
	A/G	68.1 ± 16.5	39.9 ± 22.7 *	44.5 ± 20.9	64.3 ± 44.3
		(54.0; 64.0; 80.5)	(23.6; 30.2; 65.9)	(21.5; 49.6; 62.4)	(32.9; 64.3; 95.6)
**TC/HDL ratio**	G/G	3.2 ± 0.8	4.4 ± 2.6	5.1 ± 3.8	4.4 ± 2.4
		(2.7; 3.0; 3.7)	(2.3; 3.4; 6.7)	(3.2; 4.0; 8.8)	(2.4; 4.1; 6.3)
	A/A	4.2 ± 2.3	4.9 ± 2.0	8.2 ± 4.7	6.7 ± 3.5
		(2.7; 3.0; 6.8)	(3.1; 5.4; 6.5)	(3.8; 8.5; 9.1)	(2.8; 7.6; 9.7)
	A/G	3.0 ± 0.7	4.0 ± 2.2	4.7 ± 3.2	3.7 ± 3.3
		(1.9; 2.9; 4.4)	(1.5; 5.2; 5.3)	(2.1; 3.7; 8.4)	(1.4; 3.7; 6.0)
**LDL/HDL ratio**	G/G	2.0 ± 0.7	3.1 ± 2.4 *	3.3 ± 2.9	2.8 ± 2.2
		(1.4; 1.8; 2.4)	(3.2; 2.2; 5.5)	(1.1; 3.2; 4.1)	(1.3; 2.7; 4.0)
	A/A	2.8 ± 2.1	2.8 ± 1.8	5.8 ± 4.3	7.9 ± 7.8
		(1.4; 1.8; 5.2)	(1.2; 2.6; 4.3)	(1.7; 5.8; 7.1)	(3.3; 5.5; 12.5)
	A/G	1.7 ± 0.5	2.5 ± 2.1	3.1 ± 2.2	2.4 ± 1.7
		(1.3; 1.8; 2.0)	(0.1; 3.6; 3.8)	(1.9; 1.9; 5.8)	(1.2; 2.4; 3.6)
**oxLDL/LDL ratio** **[U/g]**	G/G	56.5 ± 22.3	64.1 ± 48.3	153.4 ± 118.2 #	57.6 ± 35.5 ##
		(40.8; 53.9; 65.8)	(27.9; 50.4; 89.9)	(97.5; 105.2; 136.5)	(34.4; 46.5; 90.6)
	A/A	48.0 ± 22.8	43.6 ± 29.9	72.2 ± 26.5	68.9 ± 24.6
		(31.8; 48.0; 64.2)	(22.4; 43.6; 64.7)	(42.1; 82.3; 92.1)	(42.1; 59.7; 81.4)
	A/G	50.4 ± 30.4	42.0 ± 25.6	52.6 ± 23.3	64.5 ± 27.8
		(32.3; 46.9; 57.8)	(25.8; 52.0; 69.8)	(35.9; 50.3; 70.2)	(32.6; 62.3; 92.6)
**MDA concentration [nmol/µL]**	G/G	0.9 ± 0.6	2.2 ± 0.3 */**	2.6 ± 0.6	3.1 ± 1.0 #
		(0.3; 0.7; 1.3)	(2.0; 2.2; 2.4)	(2.2; 2.6; 3.0)	(2.5; 3.0; 3.5)
	A/A	1.0 ± 1.0	2.7 ± 0.5 *	2.7 ± 0.5	2.9 ± 0.4
		(0.4; 0.5; 2.2)	(2.5; 2.8; 3.0)	(2.5; 2.6; 2.8)	(2.5; 3.2; 3.2)
	A/G	0.7 ± 0.7	1.8 ± 0.6 */**	2.7 ± 0.7	2.7 ± 0.9
		(0.3; 0.6; 1.0)	(1.4; 1.8; 2.2)	(2.2; 2.7; 3.2)	(2.1; 2.7; 3.3)

Values shown as mean and SD (1st quartile. median. 3rd quartile). * *p* < 0.05 compared to healthy subjects with the same genotype, ** *p* < 0.05 compared to subjects with the A/A genotype, *** *p* < 0.05 compared to the G/G genotype, ^#^ *p* < 0.05 compared to the 1st day, ^##^ *p* < 0.05 compared to the 3rd day.

**Table 6:** PON1 activities, its concentration, the concentration of apoA-I, lipid profile, and selected markers of pro/antioxidative balance in the context of genotypic variability of SNP +83 bp in the *APOA1* gene in the group of healthy subjects and AP patients.

Parameters	Genotypes	Healthy Subjects (*n* = 51)	The Patients with Acute Pancreatitis (*n* = 44)		
			1st Day of Hospitalization	3rd Day of Hospitalization	7th Day of Hospitalization
**PON1**(**P**) **activity** **[U/L]**	C/C	251.0 ± 142.0	61.4 ± 29.7 *	56.9 ± 40.6	36.5 ± 16.5 #
		(147.2; 179.9; 389.1)	(42.8; 59.0; 77.8)	(31.8; 45.8; 77.5)	(36.3; 33.3; 50.4)
	T/C	236.1 ± 81.9	89.1 ± 47.5 *	156.1 ± 58.6 **	132.2 ± 28.8 **
		(178.3; 237.9; 295.6)	(55.1; 90.7; 123.1)	(102.5; 147.3; 218.7)	(99.7; 142.2; 154.7)
PON1(A) **activity** **[U/L]**	C/C	115.5 ± 42.6	104.0 ± 57.3	97.3 ± 49.3	80.1 ± 49.6
		(83.2; 114.6; 145.2)	(54.2; 108.9; 136.2)	(51.2; 101.3; 128.4)	(36.1; 74.6; 109.8)
	T/C	98.3 ± 52.1	137.1 ± 42.4	148.1 ± 12.6 **	141.3 ± 22.8 **
		(85.8; 93.9; 141.2)	(104.6; 146.6; 169.5)	(138.1; 144.0; 162.3)	(116.2; 146.6; 160.9)
**PON1**(**L**) **activity** **[U/L]**	C/C	8.7 ± 3.3	5.3 ± 3.1 *	4.9 ± 2.8	3.8 ± 2.2 #/##
		(6.4; 8.7; 10.6)	(3.5; 4.5; 6.2)	(3.0; 4.3; 5.8)	(2.6; 3.2; 4.1)
	T/C	6.6 ± 3.4	6.3 ± 1.0	7.1 ± 1.9	6.2 ± 0.8 **
		(5.5; 5.8; 10.4)	(5.5; 6.4; 7.1)	(5.8; 6.3; 9.3)	(5.5; 6.0; 7.1)
**PON1 concentration [µg/mL]**	C/C	23.7 ± 6.6	15.9 ± 7.3 *	12.3 ± 4.9 #	10.2 ± 6.0 #
		(18.5; 23.8; 28.3)	(14.7; 15.9; 17.3)	(9.8; 12.1; 14.5)	(6.8; 8.6; 10.5)
	T/C	24.8 ± 11.9	24.2 ± 1.3 **	12.1 ± 1.6 #	19.3 ± 1.4 **/#
		(11.2; 29.8; 33.2)	(23.3; 24.2; 25.1)	(10.9; 12.1; 13.2)	(18.3; 19.3; 20.3)
**ApoA-I concentration ** **[g/L]**	C/C	1.4 ± 0.4	0.9 ± 0.5 *	0.7 ± 0.6	0.4 ± 0.2 #
		(1.0; 1.3; 1.6)	(0.6; 0.7; 1.2)	(0.3; 0.5; 1.0)	(0.2; 0.4; 0.5)
	T/C	1.4 ± 0.2	0.7 ± 0.6 *	0.4 ± 0.2	0.9 ± 0.7
		(11.2; 29.8; 33.2)	(0.3; 0.5; 1.0)	(0.3; 0.4; 0.5)	(0.4; 0.7; 1.5)
**HDL concentration [mg/dL]**	C/C	64.8 ± 15.1	48.1 ± 20.7 *	42.5 ± 27.9	48.8 ± 26.3
		(52.0; 63.0; 75.0)	(28.8; 45.9; 66.4)	(18.3; 32.1; 64.8)	(26.5; 46.7; 62.8)
	T/C	64.3 ± 10.7	39.8 ± 18.6 *	46.8 ± 22.1	56.2 ± 34.3
		(56.0; 61.5; 74.0)	(26.9; 34.7; 52.6)	(21.5; 56.6; 62.3)	(32.9; 40.0; 95.6)
**TC/HDL ratio**	C/C	3.1 ± 0.7	4.2 ± 2.3 *	5.6 ± 3.5	4.9 ± 2.8
		(2.6; 2.9; 3.7)	(2.3; 3.4; 6.5)	(2.8; 4.4; 8.8)	(2.5; 4.1; 6.7)
	T/C	3.3 ± 0.5	5.5 ± 2.6 *	4.3 ± 3.6	4.2 ± 2.5
		(2.7; 3.4; 3.7)	(3.8; 5.3; 7.1)	(2.1; 2.3; 8.4)	(1.4; 5.3; 6.0)
**LDL/HDL ratio**	C/C	1.8 ± 0.6	2.6 ± 2.1	4.1 ± 3.4 #	3.1 ± 2.3
		(1.4; 1.7; 2.2)	(1.1; 2.0; 3.8)	(1.7; 3.4; 5.9)	(1.4; 2.7; 4.4)
	T/C	2.0 ± 0.4	3.6 ± 1.7 *	2.9 ± 2.5	2.8 ± 1.4
		(1.6; 2.1; 2.2)	(2.5; 3.7; 4.6)	(1.1; 1.8; 5.7)	(1.2; 3.6; 3.6)
**oxLDL/LDL ratio** **[U/g]**	C/C	52.2 ± 21.3	60.7 ± 47.0	134.5 ± 113.0 #	60.3 ± 40.4 ##
		(39.0; 50.8; 63.3)	(27.7; 45.2; 77.3)	(81.4; 97.5; 136.5)	(25.9; 62.5; 94.7)
	T/C	67.6 ± 46.6	96.2 ± 64.8	117.8 ± 82.4	46.2 ± 34.8
		(37.1; 50.8; 98.1)	(50.4; 96.2; 142.0)	(64.3; 110.5; 126.9)	(20.4; 45.7; 88.9)
**MDA concentration [nmol/µL]**	C/C	0.8 ± 0.6	2.4 ± 0.4 *	2.6 ± 0.6	3.1 ± 1.0 #
		(1.7; 2.1; 2.2)	(2.1; 2.4; 2.6)	(2.2; 2.6; 3.0)	(2.5; 3.0; 3.4)
	T/C	0.9 ± 0.9	2.0 ± 0.4 *	2.6 ± 0.5	2.8 ± 0.7
		(0.2; 0.3; 1.4)	(1.7; 2.1; 2.2)	(2.2; 2.5; 3.2)	(2.1; 3.0; 3.3)

Values shown as mean and SD (1st quartile. median. 3rd quartile). * *p* < 0.05 compared to healthy subjects with the same genotype. ** *p* < 0.05 compared to the subjects with the C/C genotype, ^#^ *p* < 0.05 compared to the first day, ^##^ *p* < 0.05 compared to the third day.

**Table 7:** Correlation coefficients for the group of healthy subjects and AP patients.

Healthy Subjects		
Genotypes for SNP rs662 in the *PON1* Gene	Correlated Parameters	Correlation Coefficients
TT	PON1(P) activity–PON1(A) activity	r = 0.6058 *p* = 0.002
	PON1(P) activity–PON1(L) activity	r = 0.4195 *p* = 0.041
	PON1(A) activity–PON1(L) activity	r = 0.8219 *p* < 0.0001
TC	PON1(A) activity–PON1(L) activity	r = 0.9768 *p* = 0.001
Genotypes for SNP −75 bp in the *APOA1* gene	**Correlated parameters**	**Correlation coefficients**
GG	PON1(A) activity–PON1(L) activity	r = 0.8265 *p* < 0.0001
AG	PON1(A) activity–PON1(L) activity	r = 0.8353 *p* = 0.003
	PON1(P) activity–MDA concentration	r = 0.7638 *p* = 0.010
AA	PON1(P) activity–MDA concentration	r = 0.99981 *p* = 0.001
Genotypes for SNP +83 bp in the *APOA1* gene	**Correlated parameters**	**Correlation coefficients**
CC	PON1(P) activity–PON1(A) activity	r = 0.4840 *p* = 0.011
	PON1(A) activity–PON1(L) activity	r = 0.8174 *p* < 0.0001
	PON1(P) activity–MDA concentration	r = 0.4264 *p* = 0.027
**AP patients**		
Genotypes for SNP rs662 in the *PON1* gene	**Correlated parameters**	**Correlation coefficients**
TT	MDA concentration–the value of LDL/HDL ratio	r = 0.9152 *p* = 0.029
TC	PON1(A) activity–PON1(L) activity	r = 0.9999 *p* = 0.008
Genotypes for SNP −75 bp in the *APOA1* gene	**Correlated parameters**	**Correlation coefficients**
GG	PON1(A) activity–PON1(L) activity	r = 0.8797 *p* = 0.021
AG	PON1(L) activity–the value of TC/HDL ratio	r = −0.998 *p* = 0.039
AA	PON1(P) activity–PON1(L) activity	r = 0.8432 *p* = 0.035
	PON1 concentration–PON1(L) activity	r = 0.9993 *p* = 0.023
	ApoA-I concentration–MDA concentration	r = −0.9842 *p* = 0.012
Genotypes for SNP +83 bp in the *APOA1* gene	**Correlated parameters**	**Correlation coefficients**
CC	PON1(A) activity–PON1(L) activity	r = 0.8772 *p* = 0.022
	PON1 concentration–the value of TC/HDL ratio	r = 0.8503 *p* = 0.032

## Data Availability

Not applicable.

## Supplementary Materials

The following supporting information can be downloaded at: https://www.mdpi.com/article/10.3390/genes13111968/s1, Figure S1: The activities of PON1, the concentrations of PON1, apoA-I and HDL in context of genotypic variability of rs662 in the *PON1* gene in the group of smoking AP patients; Figure S2: The activities of PON1, the concentrations of PON1, apoA-I and HDL in context of genotypic variability of SNP +83 bp in *APOAI* gene in the group of non-smoking and smoking AP patients; Table S1: The activities of PON1, its concentration, the concentration of apoA-I, lipid profile parameters and selected markers of pro/antioxidative balance in context of genotypic variability of SNP rs662 in the *PON1* gene and tobacco smoke exposure in the group of healthy subjects; Table S2: The activities of PON1, its concentration, the concentration of apoA-I, lipid profile parameters and selected markers of pro/antioxidative balance in context of genotypic variability of SNP -75 bp in the *APOA1* gene and tobacco smoke exposure in the group of healthy subjects; Table S3: The activities of PON1, its concentration, the concentration of apoA-I, lipid profile parameters, and selected markers of pro/antioxidative balance in context of genotypic variability of SNP −75 bp in the *APOA1* gene and tobacco smoke exposure in the group of AP patients; Table S4: The activities of PON1, its concentration, the concentration of apoA-I, lipid profile parameters and selected markers of pro/antioxidative balance in context of genotypic variability of SNP +83 bp in the *APOA1* gene and tobacco smoke exposure in the group of healthy subjects; Table S5: The association of haplotype for examined SNPs with acute pancreatitis; Table S6: Linkage disequilibrium analysis.
