# Supplementary material for: Importance of Polymorphisms in the Gene of Paraoxonase-1 (SNP rs662) and Apolipoprotein A-I (SNP rs670 and rs5069) in Non-Smoking and Smoking Healthy Subjects and Patients with Acute Pancreatitis
Source: Genes (Basel). 2022 Oct 28;13(11):1968. doi: 10.3390/genes13111968 (PMC9690293; doi:10.3390/genes13111968)
Supplement: Supplementary file 1 [file genes-13-01968-s001.zip › genes-1955888-supplementary.pdf]

Table S1. The activities of PON1, its concentration, the concentration of apoA-I, lipid profile parameters and selected markers of pro/antioxidative balance in context of genotypic variability of SNP rs662 in the *PON1* gene and tobacco smoke exposure in the group of healthy subjects.

		**Healthy subjects**		
	**Genotypes**	**Non-smokers (n=23)**	**Smokers (n=28)**	p
**PON1(P) activity [U/l]**	T/T	223.0 ± 117.1 (156.0; 170.7; 252.9)	250.2 ± 163.5 (144.2; 179.7; 336.2)	0.5498
	T/C	314.3 ± 138.3 (250.7; 288.9; 428.5)	410.5 ± 255.5 (229.8; 410.5; 591.1)	0.5251
**PON1(A) activity [U/l]**	T/T	112.4 ± 36.3 (83.4; 118.8; 131.3)	111.6 ± 51.6 (80.7; 107.2; 159.3)	0.9549
	T/C	103.8 ± 11.4 (100.3; 106.4; 112.6)	164.9 ± 26.9 * (145.9; 164.9; 183.9)	**0.0057**
**PON1(L) activity [U/l]**	T/T	8.0 ± 3.2 (5.9; 8.0; 9.4)	8.8 ± 3.6 (6.7; 9.3; 10.6)	0.4492
	T/C	6.1 ± 1.1 (5.5; 5.8; 6.4)	11.8 ± 2.4 * (10.1; 11.8; 13.5)	**0.0060**
**PON1 concentration [μg/ml]**	T/T	23.3 ± 7.4 (20.4; 25.4; 27.6)	23.6 ± 7.1 (17.8; 22.9; 28.3)	0.8991
	T/C	25.1 ± 8.1 (18.6; 25.6; 31.7)	25.8 ± 4.5 (22.6; 25.8; 29.0)	0.9199
**ApoA-I concentration [g/l]**	T/T	1.3 ± 0.4 (1.1; 1.3; 1.6)	1.4 ± 0.4 (1.0; 1.3; 1.6)	0.7258
	T/C	1.6 ± 0.4 (1.4; 1.6; 1.7)	1.6 ± 0.2 (1.5; 1.6; 1.8)	0.9228
**HDL concentration [mg/dl]**	T/T	74.0 ± 14.7 (62.0; 73.5; 82.0)	56.7 ± 10.7 * (47.5; 54.5; 64.5)	**0.0001**
	T/C	70.6 ± 11.7 (62.0; 74.0; 81.0)	64.0 ± 7.1 (59.0; 64.0; 69.0)	0.5021
**CH/HDL ratio**	T/T	2.9 ± 0.9 (2.4; 2.7; 3.0)	3.6 ± 0.9 * (2.9; 3.5; 3.9)	**0.0168**
	T/C	3.0 ± 0.7 (2.5; 2.7; 3.7)	2.7 ± 0.4 (2.4; 2.7; 3.0)	0.5940
**LDL/HDL ratio**	T/T	1.6 ± 0.7 (1.2; 1.5; 1.9)	2.2 ± 0.8 * (1.7; 2.1; 2.5)	**0.0167**
	T/C	1.8 ± 0.6 (1.3; 1.6; 2.3)	1.5 ± 0.4 (1.2; 1.5; 1.8)	0.6154
**oxLDL/LDL ratio [U/g]**	T/T	52.1 ± 23.5 (39.0; 48.7; 61.0)	59.3 ± 28.6 (40.8; 54.4; 71.9)	0.4690
	T/C	43.4 ± 16.8 (32.3; 41.2; 54.5)	47.6 ± 22.2 (31.8; 47.6; 63.3)	0.8054
**MDA concentration [nmol/μl]**	T/T	0.8 ± 0.7 (0.3; 0.6; 1.5)	0.7 ± 0.6 (0.3; 0.6; 1.1)	0.5723
	T/C	1.1 ± 0.7 (0.9; 1.1; 1.2)	1.3 ± 1.4 (0.3; 1.3; 2.2)	0.8722

Values shown as mean and SD (1st quartile, median, 3rd quartile), * p<0.05 compared to non-smokers with the same genotype

Figure S1. The activities of PON1, the concentrations of PON1, apoA-I and HDL in context of genotypic variability of rs662 in *PON1* gene in the group of smoking AP patients.

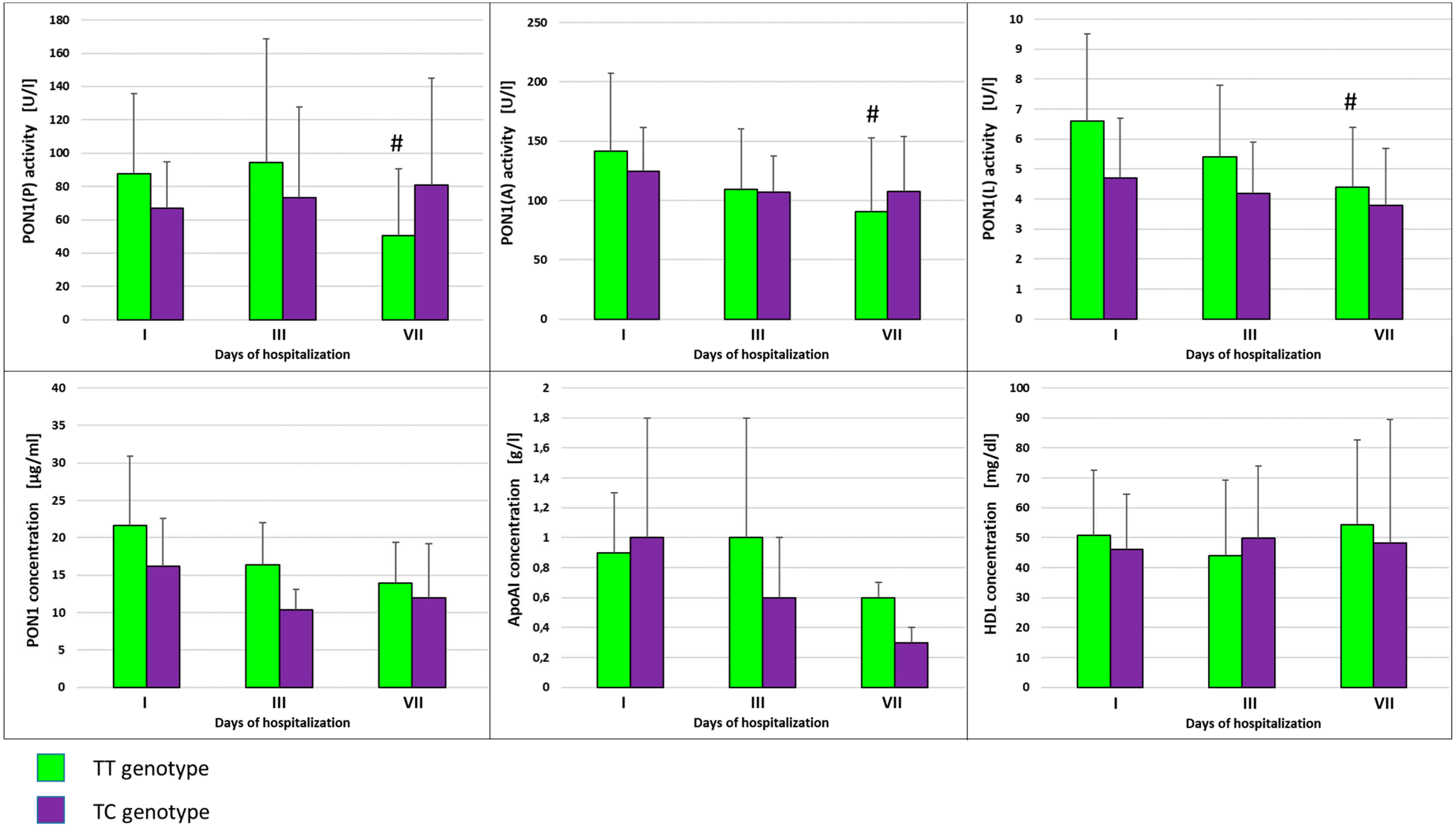

Table S2. The activities of PON1, its concentration, the concentration of apoA-I, lipid profile parameters and selected markers of pro/antioxidative balance in context of genotypic variability of SNP -75 bp in the *APOA1* gene and tobacco smoke exposure in the group of healthy subjects.

		**Healthy subjects**		
	**Genotypes**	**Non-smokers (n=23)**	**Smokers (n=28)**	p
**PON1(P) activity [U/l]**	GG	250.2 ± 132.2<br>(149.2; 206.0; 352.7)	268.0 ± 176.3<br>(145.1; 179.7; 389.1)	0.7662
	AA	163.1 ± 89.1<br>(108.2; 189.0; 244.8)	367.7 ± 316.0 *<br>(144.2; 367.7; 591.1)	**0.0042**
	AG	242.0 ± 126.1<br>(156.0; 190.2; 288.9)	208.2 ± 104.5<br>(132.7; 183.3; 245.7)	0.5902
**PON1(A) activity [U/l]**	GG	107.9 ± 29.7<br>(84.6; 109.5; 127.7)	108.0 ± 55.8<br>(67.5; 107.2; 150.3)	0.9924
	AA	122.6 ± 32.4<br>(96.7; 128.5; 142.3)	145.2 ± 1.0 *<br>(144.5; 145.2; 145.9)	**0.0336**
	AG	112.5 ± 38.4<br>(85.4; 115.3; 126.2)	129.9 ± 42.8<br>(94.7; 131.7; 170.3)	0.4131
**PON1(L) activity [U/l]**	GG	6.7 ± 1.6<br>(5.8; 6.4; 8.1)	8.6 ± 3.6<br>(6.2; 9.3; 11.0)	0.1036
	AA	9.4 ±3.8<br>(7.4; 10.8; 12.4)	10.4 ± 0.5<br>(10.1; 10.4; 10.8)	0.3639
	AG	8.5 ± 4.0<br>(5.2; 8.0; 11.3)	10.2 ± 4.0<br>(8.3; 9.3; 10.6)	0.4313
**PON1 concentration [μg/ml]**	GG	27.1 ± 6.2<br>(25.4; 27.6; 30.4)	24.9 ± 6.2<br>(18.5; 22.9; 29.9)	0.4308
	AA	28.4 ± 7.1<br>(22.4; 26.8; 33.0)	29.0 ± 5.8<br>(25.4; 29.3; 32.1)	0.9989
	AG	18.7 ± 6.5<br>(15.8; 21.1; 23.8)	18.9 ± 8.4<br>(11.2; 17.5; 27.9)	0.9714
**ApoA-I concentration [g/l]**	GG	1.4 ± 0.5<br>(1.1; 1.3; 1.8)	1.5 ± 0.4<br>(1.2; 1.5; 1.6)	0.6836
	AA	2.1 ± 0.5<br>(1.7; 2.0; 2.3)	1.2 ± 0.4 *<br>(1.0; 1.2; 1.5)	**0.0001**
	AG	1.3 ± 0.4 **<br>(1.2; 1.3; 1.4)	1.1 ± 0.2<br>(1.0; 1.1; 1.2)	0.4266
**HDL concentration [mg/dl]**	GG	68.5 ± 12.3 **<br>(59.5; 69.0; 77.5)	58.8 ± 11.7 *<br>(46.0; 60.5; 69.0)	**0.0384**
	AA	94.0 ± 14.3<br>(87.8; 82.3; 104.0)	54.0 ± 7.1 *<br>(49.0; 54.0; 59.0)	**<0.0001**
	AG	76.9 ± 14.0<br>(64.0; 79.5; 81.0)	53.5 ± 7.2 *<br>(49.0; 51.5; 60.0)	**0.0020**
**CH/HDL ratio**	GG	3.1 ± 1.0<br>(2.5; 2.8; 3.5)	3.4 ± 0.7 **<br>(2.9; 3.3; 3.8)	0.3892
	AA	2.7 ± 0.4<br>(2.4; 2.8; 3.1)	4.9 ± 2.7<br>(3.0; 4.9; 6.8)	0.6230
	AG	2.7 ± 0.5<br>(2.5; 2.7; 3.0)	3.5 ± 0.6 */**<br>(3.1; 3.5; 3.9)	**0.0206**
**LDL/HDL ratio**	GG	1.8 ± 0.7<br>(1.2; 1.6; 2.2)	2.1 ± 0.6 **<br>(1.5; 2.0; 2.5)	0.2940
	AA	1.4 ± 1.0<br>(0.8; 1.8; 2.1)	3.5 ± 2.4 *<br>(1.8; 3.5; 5.2)	**0.0003**
	AG	1.5 ± 0.5	2.0 ± 0.4 **	0.0641

		(1.2; 1.5; 1.9)	(1.9; 2.0; 2.1)	
**oxLDL/LDL ratio [U/g]**	GG	56.0 ± 26.6 (39.2; 46.6; 65.8)	56.9 ± 19.7 (42.2; 55.1; 67.6)	0.9242
	AA	64.2 ± 17.1 (49.2; 62.0; 71.8)	31.8 ± 19.2 * (28.1; 45.1; 48.3)	**<0.0001**
	AG	41.9 ± 15.0 (32.3; 41.8; 54.6)	67.4 ± 47.9 (38.5; 55.8; 96.2)	0.1830
**MDA concentration [nmol/µl]**	GG	1.0 ± 0.5 (0.6; 0.9; 1.4)	0.8 ± 0.7 (0.2; 0.5; 1.2)	0.3778
	AA	0.5 ± 0.4 (0.3; 0.8; 0.9	1.3 ± 1.3 * (0.4; 1.3; 2.2)	**0.0066**
	AG	0.9 ± 0.8 (0.2; 0.4; 1.5)	0.6 ± 0.3 (0.3; 0.6; 0.7)	0.4321

Values shown as mean and SD (1st quartile, median, 3rd quartile), * $p<0.05$ compared to non-smokers with the same genotype, ** $p<0.05$ compared to individuals with the AA genotype.

Table S3. The activities of PON1, its concentration, the concentration of apoA-I , lipid profile parameters and selected markers of pro/antioxidative balance in context of genotypic variability of SNP -75 bp in the *APOA1* gene and tobacco smoke exposure in the group of AP patients.

Parameters in plasma	Genotype	1st day of hospitalization		3rd day of hospitalization		7th day of hospitalization	
		Non-smokers (n=17)	Smokers (n=27)	Non-smokers (n=17)	Smokers (n=27)	Non-smokers (n=17)	Smokers (n=27)
**PON1(P) activity**	G/G	42.7 ± 19.2	81.3 ± 46.3	27.0 ± 13.6	78.6 ± 62.7	34.2 ± 13.7	48.9 ± 41.0
**[U/l]**		(30.3; 42.2; 43.4)	(59.0; 73.5; 80.1)	(11.6; 32.3; 37.2)	(35.6; 52.0; 102.5)	(26.3; 26.4; 50.1)	(27.5; 41.9; 52.3)
	A/A	70.6 ± 72.2	56.6 ± 21.8	53.2 ± 23.2	51.9 ± 37.8	64.7 ± 32.2	28.8 ±13.3
		(19.5; 70.6; 121.7)	(44.2; 47.2; 68.9)	(41.8; 65.3; 73.4)	(27.1; 39.3; 76.7)	(48.2; 74.2; 79.1)	(19.4; 28.8; 38.2)
	A/G	144.5 ± 60.8 *	101.7 ± 32.4	218.7± 150.8 *	151.1 ± 5.5**	99.7 ± 34.7	154.0 ± 72.4 *
		(93.2; 158.2; 184.3)	(78.2; 121.2; 129.4)	(110.2; 268.4; 296.2)	(147.3;151.1; 155.0)	(66.7; 96.2; 117.4)	(89.3; 167.2; 198.4)
**PON1(A) activity**		97.0 ± 61.4	109.9 ±45.6	77.0 ± 63.9	104.7 ± 48.4	87.6 ± 54.1	93.7 ± 56.9
**[U/l]**	G/G	(38.6; 104.1; 147.3)	(68.0; 121.3; 136.2)	(20.9; 66.4; 154.4)	(53.7; 116.9; 150.4)	(30.6; 94.0; 138.3)	(58.9; 74.6; 128.7)
		83.7 ± 52.9	120.1 ±93.6	83.5 ± 35.5	113.5 ± 61.7	72.9 ± 52.2	54.8 ± 45.5
	A/A	(28.9; 87.6; 134.5)	(54.3; 112.3; 185.9)	(43.0; 98.2; 109.2)	(69.1; 103.4; 157.9)	(36.1; 72.9; 109.8)	(22.6; 54.8; 86.9)
		80.8 ±53.2	165.2 ±12.7 *. **/$	138.1 ± 54.2 */**	141.4 ± 3.7	116.2 ± 44.3 **	160.0 ± 58.7 */**
	A/G	(36.4; 90.4; 128.2)	(156.3; 165.2; 174.2)	(87.4; 122.1; 11.3)	(138.8; 141.4; 144.0)	(78.2; 110.4; 148.7)	(104.7; 154.2; 197.4)
**PON1(L) activity**	G/G	4.4 ±3.7	4.5 ±0.9	3.6 ± 2.6 **	4.4 ±1.3	3.6 ± 2.3	3.7 ± 1.9
**[U/l]**		(1.1; 4.8; 6.0)	(4.1; 4.4; 5.0)	(0.6; 3.4; 6.9)	(3.1; 4.5; 5.7)	(1.6; 2.9; 6.1)	(2.5; 3.2; 4.6)
	A/A	7.9 ±3.2	4.5 ± 3.1	6.8 ± 3.4	4.4 ± 2.6	6.5 ± 3.4	2.9 ± 0.3
		(4.1; 9.6; 9.9)	(2.2; 4.0; 6.9)	(3.0; 7.7; 9.6)	(2.3; 3.9; 6.5)	(4.1; 6.5; 8.8)	(2.8; 2.9; 3.1)
	A/G	6.8 ± 4.2	10.2 ± 4.0 **	9.3 ± 3.6 *	9.3 ± 5.0 *	7.1 ± 3.3 *	5.9 ± 3.2 *. **
		(5.4; 7.8; 9.3)	(7.4; 10.2; 13.1)	(6.8; 9.2; 11.3)	(5.8; 9.3; 12.9)	(4.5; 7.8; 8.9)	(4.8; 6.1; 7.2)
**PON1**	G/G	10.0 ± 6.9	17.6 ± 3.3 $	9.0 ± 5.3	14.4 ± 4.4	10.8 ± 10.0	11.8 ± 5.1 #
**concentration**		(2.1; 13.0; 15.0)	(15.8; 16.4; 18.4)	(2.9; 12.1; 12.1)	(0.2; 13.9; 18.7)	(3.6; 6.6; 22.2)	(8.6; 9.6; 18.1)
**[μg/ml]**	A/A	12.9 ± 5.4	20.1 ± 13.7	11.6 ± 4.1	10.4 ± 3.3	10.5 ± 4.9	7.2 ± 5.1 *
		(7.8; 13.5; 16.9)	(7.8; 17.6; 34.8)	(8.3; 12.8; 15.5)	(7.1; 10.4; 13.7)	(6.8; 12.7; 14.3	(3.9; 7.9; 11.8)
	A/G	14.4 ± 6.1	23.3 ± 11.2 $	12.2 ± 3.8	10.9 ± 4.0 #	(11.6 ± 5.8	12.9 ± 6.4 **. #
		(9.2; 16.8; 19.3)	(12.5; 25.2; 32.5)	(8.9; 13.4; 16.2)	(7.2; 11.3; 14.1)	(7.2; 12.8; 15.9)	(6.8; 13.6; 18.0)
**ApoA-I**	G/G	0.5 ± 0.4	1.0 ± 0.4 $	0.7 ± 0.4	0.9 ± 0.6	0.6 ± 0.3	0.6 ± 0.2
**concentration**		(0.2; 0.3; 0.6)	(0.6; 1.0; 1.4)	(0.5; 0.7; 0.9)	(0.5; 0.9; 1.3)	(0.4; 0.6; 0.7)	(0.4; 0.6; 0.7)
**[g/l]**	A/A	1.1 ± 0.7 *	1.0 ± 0.8	0.5 ± 0.3 #	0.5 ± 0.5	0.5 ± 0.4 #/*	0.3 ± 0.3 *
		(0.6; 1.1; 1.6)	(0.5; 0.7; 1.4)	(0.3; 0.5; 0.8)	(0.2; 0.4; 0.8)	(0.3; 0.5; 0.7)	(0.1; 0.3; 0.5)
	A/G	1.9 ± 0.7 *. **	0.6 ± 0.2 $	0.4 ± 0.3 #	1.2 ± 1.0 $	1.2 ± 0.6 #/ ##	0.7 ± 0.4 **/$

		(1.5; 2.0; 2.5)	(0.4; 0.6; 0.7)	(0.4; 0.5; 0.6)	(0.5; 1.2; 1.9)	(0.9; 1.4; 1.7)	(0.6; 0.8; 0.9)
**HDL concentration**	G/G	45.0 ± 17.9	52.9 ± 24.1	44.1 ± 33.4	49.3 ± 27.9	38.1 ± 11.6	61.8 ± 25.1
**[mg/dl]**		(30.6; 39.2; 56.9)	(27.5; 53.4; 77.1)	(1.5; 30.6; 93.7)	(18.3; 56.1; 74.9)	(25.0; 42.3; 47.1)	(40.0; 62.8; 85.0)
	A/A	26.4 ± 3.9	48.7 ± 12.4	11.7 ± 3.4 #	39.3 ± 26.6	37.6 ± 23.4	18.9 ± 3.5 #/*
		(23.6; 26.4; 29.2)	(41.5; 44.1; 55.8)	(9.3; 11.7; 14.1)	(23.6; 31.4; 55.0)	(11.1; 46.3; 55.4)	(16.5; 18.9; 21.3)
	A/G	30.2 ± 8.1	44.8 ± 29.9	21.5 ± 7.2	56.0 ± 9.0	32.9 ± 9.4	46.2 ± 10.1 **
		(22.9; 32.4; 36.1)	(23.6; 44.8; 65.9)	(15.4; 23.0; 26.2)	(49.6; 56.0; 62.4)	(26.2; 34.2; 38.0)	(39.2; 49.3; 54.0)

Values shown as mean and SD (1st quartile, median, 3rd quartile), * $p<0.05$ compared to subjects with the G/G genotype, ** $p<0.05$ compared to subjects with the A/A genotype, # $p<0.05$ compared to the 1st day of hospitalization, ## $p<0.05$ compared to the 3rd day of hospitalization, $ $p<0.05$ compared to non-smokers

Table S4. The activities of PON1, its concentration, the concentration of apoA-I, lipid profile parameters and selected markers of pro/antioxidative balance in context of genotypic variability of SNP +83 bp in the *APOA1* gene and tobacco smoke exposure in the group of healthy subjects.

		**Healthy subjects**		
	**Genotypes**	**Non-smokers (n=23)**	**Smokers (n=28)**	p
**PON1(P) activity [U/l]**	CC	251.3 ± 127.3 (156.5; 200.7; 288.9)	259.5 ± 183.5 (132.7; 177.4; 397.6)	0.8642
	TC	154.6 ± 33.6 (130.8; 154.6; 178.3)	276.9 ± 64.5 (237.9; 293.1; 315.9)	0.0725
**PON1(A) activity [U/l]**	CC	111.4 ± 33.6 (85.4; 113.9; 126.2)	119.2 ± 49.9 (80.7; 122.2; 163.5)	0.5500
	TC	100.6 ± 20.8 (85.8; 100.6; 115.3)	97.5 ± 62.9 (85.8; 93.9; 141.2)	0.9509
**PON1(L) activity [U/l]**	CC	7.7 ± 3.0 (5.8; 7.0; 9.4)	9.6 ± 3.3 (8.3; 9.5; 11.5)	0.0600
	TC	6.6 ± 1.6 (5.5; 6.6; 7.7)	6.6 ± 4.0 (5.5; 5.8; 10.4)	0.9861
**PON1 concentration [μg/ml]**	CC	23.7 ± 7.3 (20.4; 25.4; 28.4)	23.7 ± 5.7 (18.2; 22.8; 28.1)	0.9975
	TC	23.6 ± 8.1 (19.4; 25.1; 30.4)	24.8 ± 11.9 (11.2; 29.9; 33.3)	0.6826
**ApoA-I concentration [g/l]**	CC	1.4 ± 0.5 (1.2; 1.4; 1.8)	1.4 ± 0.5 (1.0; 1.2; 1.6)	0.8920
	TC	1.2 ± 0.2 (1.1; 1.2; 1.4)	1.4 ± 0.2 (1.3; 1.6; 1.6)	0.3753
**HDL concentration [mg/dl]**	CC	72.9 ± 14.5 (62.0; 73.0; 81.0)	57.1 ± 11.3 * (46.0; 56.0; 66.0)	**0.0003**
	TC	77.0 ± 4.2 (74.0; 77.0; 80.0)	58.0 ± 5.1 * (54.5; 57.0; 61.5)	**0.0110**
**CH/HDL ratio**	CC	2.9 ± 0.9 (2.5; 2.7; 3.2)	3.5 ± 1.0 * (2.9; 3.2; 3.9)	**0.0498**
	TC	2.7 ± 0.0 (2.7; 2.7; 2.7)	3.6 ± 0.2 * (3.4; 3.6; 3.7)	**0.0035**
**LDL/HDL ratio**	CC	1.7 ± 0.7 (1.2; 1.5; 1.9)	2.1 ± 0.9 * (1.5; 1.9; 2.5)	**0.0493**
	TC	1.5 ± 0.0 (1.5; 1.5; 1.6)	2.2 ± 0.2 * (2.1; 2.2; 2.3)	**0.0065**
**oxLDL/LDL ratio [U/g]**	CC	49.5 ± 22.6 (39.0; 43.2; 60.4)	55.4 ± 20.1 (43.5; 55.3; 63.3)	0.4594
	TC	61.0 ± 25.6 (45.3; 62.8; 78.9)	69.8 ± 56.8 (33.4; 40.8; 135.2)	0.9049
**MDA concentration [nmol/μl]**	CC	1.0 ± 0.7 (0.5; 0.8; 1.5)	0.7 ± 0.6 (0.3; 0.6; 0.9)	0.1739
	TC	0.3 ± 0.1 (0.2; 0.3; 0.3)	1.1 ± 1.0 (0.2; 1.1; 1.4)	0.3698

Values shown as mean and SD (1st quartile, median, 3rd quartile), * $p<0.05$ compared to non-smokers with the same genotype.

Figure S2. The activities of PON1, the concentrations of PON1, apoA-I and HDL in context of genotypic variability of SNP +83 bp in *APOAI* gene in the group of non-smoking and smoking AP patients.

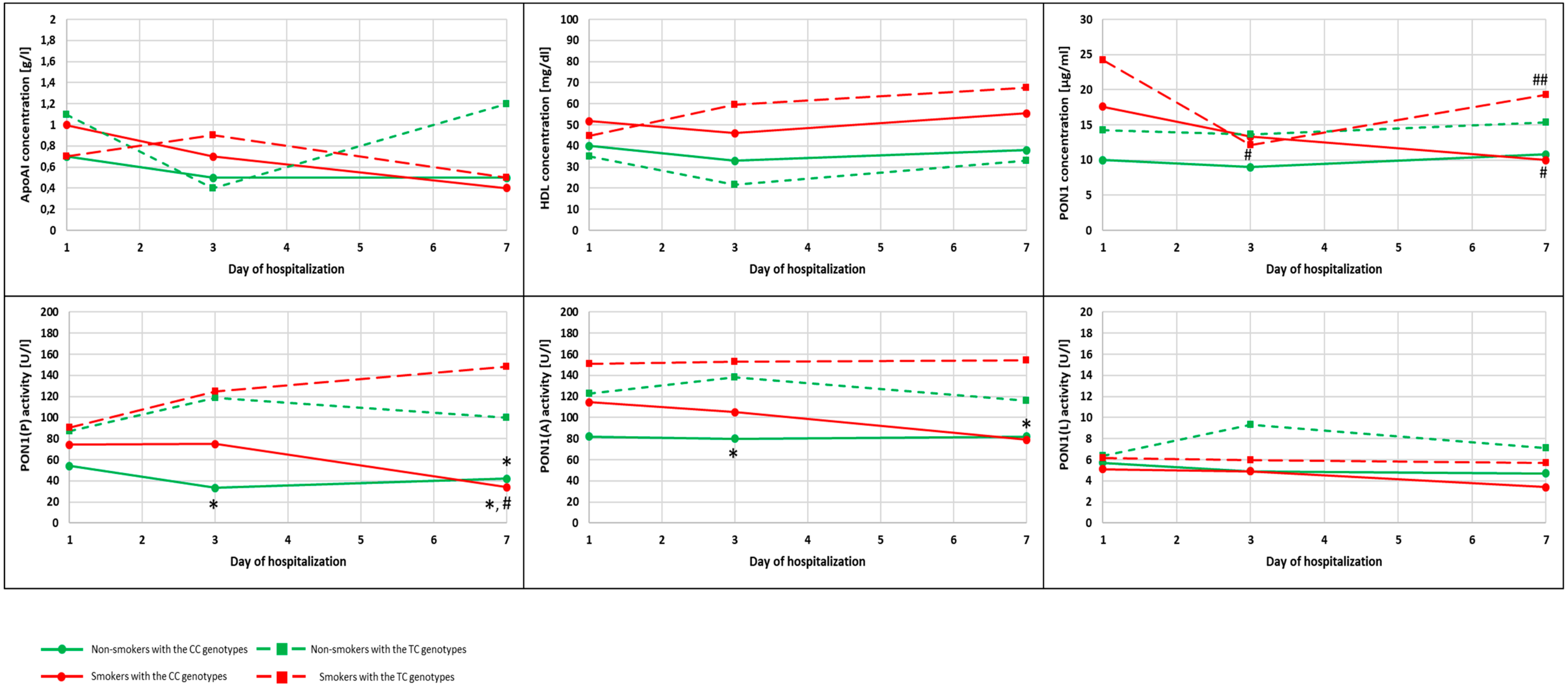

*p<0.05 compared to AP patients with the TC genotypes, # p<0.05 compared to the 1st day of hospitalization, ## p<0.05 compared to the 3rd day of hospitalization

Table S5. The association of haplotype for examined SNPs with acute pancreatitis.

rs662 (*PON1*)	rs670 (-75 G/A) (*APOA1*)	rs5069 (+83 C/T) (*APOA1*)	Frequency	OR (95% CI)	p-value
T	G	C	0.6011	1.00	-
T	A	C	0.2272	0.77 (0.40 - 1.49)	0.44
T	G	T	0.0717	0.59 (0.17 - 2.07)	0.41
T	A	T	0.0421	0.00	1.00
C	G	C	0.0357	3.37 (0.34 - 33.17)	0.3
C	A	C	0.0202	0.47 (0.05 - 4.49)	0.51

Table S6. Linkage disequilibrium analysis.

	**D statistic**		**D′ statistic**		**r statistic**		**p-values**	
	rs670 (-75 G/A) (*APOA1*)	rs5069 (+83 C/T) (*APOA1*)	rs670 (-75 G/A) (*APOA1*)	rs5069 (+83 C/T) (*APOA1*)	rs670 (-75 G/A) (*APOA1*)	rs5069 (+83 C/T) (*APOA1*)	rs670 (-75 G/A) (*APOA1*)	rs5069 (+83 C/T) (*APOA1*)
rs662 (*PON1*)	0.0055	-0.0036	0.1332	0.5433	0.0517	-0.0487	0.4758	0.5017
rs670 (-75 G/A) (*APOA1*)	-	0.0122	-	0.1481	-	0.0839	-	0.2473
